# Tilapia Piscidin 4 (TP4) Reprograms M1 Macrophages to M2 Phenotypes in Cell Models of *Gardnerella vaginalis*-Induced Vaginosis

**DOI:** 10.3389/fimmu.2021.773013

**Published:** 2021-12-02

**Authors:** Chia-Wen Liu, Bor-Chyuan Su, Jyh-Yih Chen

**Affiliations:** ^1^ Marine Research Station, Institute of Cellular and Organismic Biology, Academia Sinica, Jiaushi, Taiwan; ^2^ Department of Anatomy and Cell Biology, School of Medicine, College of Medicine, Taipei Medical University, Taipei, Taiwan; ^3^ The iEGG and Animal Biotechnology Center, The Rong Hsing Research Center for Translational Medicine, National Chung Hsing University, Taichung, Taiwan

**Keywords:** bacterial vaginosis, *Gardnerella vaginalis*, macrophage polarization, antimicrobial peptide, tilapia piscidin 4

## Abstract

*Gardnerella vaginalis* is associated with bacterial vaginosis (BV). The virulence factors produced by *G. vaginalis* are known to stimulate vaginal mucosal immune response, which is largely driven by activated macrophages. While Tilapia piscidin 4 (TP4), an antimicrobial peptide isolated from Nile tilapia, is known to display a broad range of antibacterial functions, it is unclear whether TP4 can affect macrophage polarization in the context of BV. In this study, we used the culture supernatants from *G. vaginalis* to stimulate differentiation of THP-1 and RAW264.7 cells to an M1 phenotype. The treatment activated the NF-κB/STAT1 signaling pathway, induced reactive nitrogen and oxygen species, and upregulated inflammatory mediators. We then treated the induced M1 macrophages directly with a non-toxic dose of TP4 or co-cultured the M1 macrophages with TP4-treated vaginal epithelial VK2 cells. The results showed that TP4 could not only decrease pro-inflammatory mediators in the M1 macrophages, but it also enriched markers of M2 macrophages. Further, we found that direct treatment with TP4 switched M1 macrophages toward a resolving M2c phenotype *via* the MAPK/ERK pathway and IL-10-STAT3 signaling. Conversely, tissue repair M2a macrophages were induced by TP4-treated VK2 cells; TP4 upregulated TSG-6 in VK2 cells, which subsequently activated STAT6 and M2a-related gene expression in the macrophages. In conclusion, our results imply that TP4 may be able to attenuate the virulence of *G*. *vaginalis* by inducing resolving M2c and tissue repair M2a macrophage polarizations, suggesting a novel strategy for BV therapy.

## Introduction

Bacterial vaginosis (BV) is one of the most common vaginal infectious diseases among women of reproductive age, and it is difficult to manage clinically (1, 2). BV is caused by disturbances in the vaginal microflora; lactobacilli are depleted, allowing the mucosa to become dominated by aerobic and facultative bacteria (3). Among BV-associated organisms, *Gardnerella vaginali*s is thought to play a key role in the condition (4, 5). Secretion of virulence factors is a major mechanism of *G. vaginalis* toxicity, as these factors heighten the inflammatory response, decrease the vaginal epithelial integrity, and inhibit tissue repair (6–9). These effects are largely responsible for BV symptoms and create a more permissive environment for the acquisition and spread of sexually transmitted infections, such as HIV (10). At present, antibiotic treatments are the most common therapy for BV (11); however, accumulating reports have challenged this treatment strategy. Antibiotics not only indiscriminately destroy bacteria, but they may also cause adverse effects on the host immune system, such as damage to lymphocyte DNA or reductions in the numbers and functions of macrophages and lymphocytes (12–14). Therefore, it will be beneficial to find new therapeutic options for BV that can improve the risk-benefit ratio for patients by maintaining healthy vaginal microflora and immune function.

Monocytes and macrophages are normally found in the lamina propria of vaginal mucosa and play crucial roles in the mucosal barrier, including prevention of pathogen invasion and response to a variety of microbial virulence factors (15, 16). Once bacteria-derived stimuli are detected, macrophages differentiate into the classical inflammatory (M1) macrophages through the production of the microbicidal reactive nitrogen and oxygen intermediates, and the activation of nuclear factor-κB (NFκB) and signal transducer and activator of transcription (STAT) 1. The NFκB/STAT pathway then transcriptionally activates pro-inflammatory molecules, such as interleukin (IL)-12, tumor necrosis factor (TNF)-α, and IL-1β (17). During later stages of the inflammatory response, macrophages respond to autocrine or paracrine (i.e., received from epithelial cells) signals that shift the macrophages from the M1 phenotype to alternatively activated resolving (M2c) or tissue repair (M2a) types (18). M2c macrophages frequently arise from MAPK pathway-induced IL-10-STAT3 signaling and serve to prevent excessive inflammation. On the other hand, differentiation of M2a macrophages can be driven by IL-4/13-induced STAT6 signaling, and these cells function to restore normal tissue structures (19). Recently, TNF-α-stimulated gene 6 (TSG-6), an anti-inflammatory protein detected in epithelium, has also been shown to activate STAT6 signaling in macrophages (20, 21). The polarization of macrophages into suitable types upon receipt of certain stimuli is necessary for effective tissue repair in many contexts. Dysregulation and prolonged macrophage response cause persistent inflammation and poor tissue repair seen in many diseases including BV, consequently increasing the risk of other pathogenic infections (22–24).

Antimicrobial peptides (AMPs) are a class of short, cationic and amphipathic molecules secreted by immune and epithelial cells in response to invasive bacteria and their products (25, 26). Tilapia piscidin 4 (TP4) was recently identified in Nile tilapia (*Oreochromis niloticus*) (27). Owing to its alpha-helical secondary structure, net positive charge and optimum hydrophobicity, TP4 has high activity against a broad spectrum of bacterial pathogens, and it can eradicate microbial biofilms (28, 29). In addition to its low hemolytic activity and cytotoxicity in various models, TP4 may be useful as a potential treatment for infectious diseases (30–32). Besides its antimicrobial activities, TP4 also has immunomodulatory functions that can provide a counterbalance to the production of inflammatory cytokines in bacteria-induced inflammation and enhance epidermal wound healing (31–33). However, the underlying mechanisms of this immunomodulation are still unclear.

In this study, we aimed to investigate the modulatory effect of TP4 on macrophage polarization induced by *G. vaginalis*. Using *G. vaginalis*-free culture supernatants, inflammatory M1 phenotypes were induced in THP-1 and RAW264.7 cells. We found that either treatment of TP4 directly to M1 macrophages or indirectly to vaginal epithelial VK2 cells co-cultured with the M1 macrophages could reduce the inflammatory mediators in the M1 macrophages. Further, we discovered that TP4 promoted the phenotype of resolving M2c macrophage through MAPK/ERK pathway-induced IL-10-STAT3 signaling. In the co-culture model, TP4 induced VK2 and endocervical cells to secrete TNF-α-stimulated gene 6 (TSG-6), which elicited the phenotypes of tissue repair M2a macrophages through STAT6 activation. These results suggest that TP4 may reduce the virulence of *G*. *vaginalis* by dampening the induced inflammation and promoting tissue remodeling *via* modulation of macrophage polarizations.

## Materials and Methods

### Cell Culture

The human THP-1 (BCRC 60430) and mouse RAW264.7 macrophages (BCRC 60001) were obtained from the Bioresource Collection and Research Center (BCRC) and respectively cultured in Roswell Park Memorial Institute (RPMI)-1640 medium (Gibco) and Dulbecco modified Eagle’s medium (DMEM; Gibco) supplemented with 10% fetal bovine serum (FBS; Gibco) and 10 mM HEPES (Sigma). To induce differentiation of THP-1 cells into macrophages, the cells were cultured in 100 nM phorbol 12-myristate 13-acetate (PMA; Cayman Chemical) for 48 h (34). Vaginal epithelial (VK2, ATCC# CRL-2616) and endocervical epithelial (End1, ATCC# CRL-2615) cell lines were purchased from the American Type Culture Collection (ATCC) and cultured in keratinocyte serum-free medium (KSFM; Gibco) supplemented with 0.1 ng/ml epidermal growth factor, 50 mg/ml bovine pituitary extract (ScienCell Research Laboratories), and 0.4 mM calcium chloride (Sigma). All culture media were supplemented with 100 U/ml penicillin and 100 μg/ml streptomycin (Hyclone), and cells were maintained in a humidified incubator containing 5% CO_2_ at 37°C.

### Bacterial Culture and Preparation of Bacteria-Free Culture Supernatants

*Gardnerella vaginalis* strain (ATCC# 14018) was cultured in Tryptic Soy Broth (TSB; BD Biosciences) with 5% defibrinated rabbit blood (Rockland Immunochemicals) under anaerobic conditions with the AnaeroPack system (Mitsubishi Gas Chemical Company) at 37°C for 72 h. The cultures and control medium (TSB broth) were centrifuged three times for 10 min each at 2,500 rpm at 4°C and then sterilized using a 0.22 μm syringe filter (EMD Millipore) to remove any remaining bacterial debris. The samples were stored frozen at -80°C until use. To generate the model of *G. vaginalis*-free culture supernatants (GV sup)-induced M1 macrophages, PMA-differentiated THP-1 cells and RAW264.7 cells were incubated with 5 or 10% (v/v) GV sup in RPMI-1640 and DMEM cell growth media, respectively for 24 h. For the subsequent induction of GV sup-induced THP-1 cells and RAW264.7 cells (THP-1/GV and RAW264.7/GV), 10% (v/v) GV sup in the cell growth medium was used. To produce GV sup-induced epithelial inflammation and apoptosis, VK2 cells were treated with 10% (v/v) GV sup in KSFM cell growth medium.

### Immunoblotting

Total protein lysates were prepared on ice using RIPA lysis buffer (Merck Millipore) supplemented with protease and phosphatase inhibitors cocktails (Roche). Protein concentrations were determined with the BCA Protein Assay (Pierce). Equal amounts of the boiled lysate (50 μg protein) were separated on acrylamide gels and then transferred to polyvinylidene difluoride (PVDF) membranes (Merck Millipore). After blocking with blocking solution (0.1 M phosphate buffer solution (PBS), 5% non-fat milk, 0.2% Tween-20), membranes were probed with primary antibodies (1:1000) at 4°C overnight. The following primary antibodies were used: NFκB p65 (8242; Cell Signaling Technology), phospho-NF-κB p65 (3033; Cell Signaling Technology), phospho-STAT1 (9177; Cell Signaling Technology), GAPDH (5174; Cell Signaling Technology), α-tubulin (2125; Cell Signaling Technology), β-actin (4970; Cell Signaling Technology), cleaved PARP (5625; Cell Signaling Technology), IL-10 (12163; Cell Signaling Technology), IL-10 (ab189392; Abcam), phospho-STAT3 (9145; Cell Signaling Technology), phospho-STAT6 (56554; Cell Signaling Technology), ERK (4695; Cell Signaling Technology), phospho-ERK (4370; Cell Signaling Technology), p38 (8690; Cell Signaling Technology), phospho-p38 (9211; Cell Signaling Technology), TSG-6 (sc-398307; Santa Cruz Biotechnology). Membranes were then incubated with HRP-conjugated secondary antibodies (1:10000) (GE Healthcare Amersham) for 1 h at room temperature. The blots were developed with the ECL detection system according to the manufacturer’s instructions (GE Healthcare Amersham). Blot images were scanned, and a region of interest around the band of interest was defined and measured by densiometry. Data were obtained from three independent experiments.

### Nitric Oxide (NO) Assay

The production of NO by the cells was assessed by measuring nitrite accumulated in the culture medium through a Griess reaction (35). Briefly, 100 μl of cell culture medium was mixed with 100 μl of the Griess reagent (Sigma) and incubated at room temperature for 15 min before the absorbance was measured at 540 nm in a plate reader (SpectraMax i3 Multi-Mode Microplate Reader). Nitrite levels were calculated from a standard curve with known concentrations of sodium nitrite (Sigma).

### 2’,7’-Dichlorofluorescin Diacetate (DCFDA) Assay

Intracellular reactive oxygen species (ROS) generation was assessed using the oxidation-sensitive fluorescent probe, DCFDA (Molecular Probes). Cells were incubated with DCFDA (10 μM) for 1 h at 37°C in the dark. The cells were then suspended by trypsinization and washed twice with PBS. The mean fluorescence intensity of DCFDA (excitation/emission: 490/520 nm) was analyzed by flow cytometry (Beckman Coulter, Indianapolis, IN, USA).

### Cell Surface Levels of CD80 and CD206

Cells were suspended by trypsinization, fixed with 4% paraformaldehyde (Sigma) for 15 min at room temperature, and blocked with 3% bovine serum albumin (BSA; Sigma) for 1 h on ice. Afterward, THP-1 cells were incubated with anti-human CD80-APC (565157, BD Biosciences) or anti-human CD206-FITC (551135, BD Biosciences). RAW264.7 cells were incubated with anti-mouse CD80-APC (560016, BD Biosciences) or anti-mouse CD206-FITC (MA5-16870, Invitrogen) for 1 h on ice and washed three times with PBS containing 0.05% BSA. Cells were re-suspended in PBS and then subjected to flow cytometry analysis (Beckman Coulter, Indianapolis, IN, USA).

### Quantitative Real-Time PCR (qRT-PCR) Analysis

Total RNA was extracted using TRIzol Reagent (Invitrogen), and 1 μg total RNA from each sample was reverse transcribed into cDNA with RT-PCR Quick Master Mix (Toyobo) according to the manufacturer’s instructions. The qRT-PCR was performed using StepOne Plus Real-Time QPCR System (Applied Biosystems) and set up in MicroAmp fast 96 well reaction plate (Applied Biosystems). The reaction mixture was prepared in a final volume of 20 μl per reaction. Briefly, 2 μl of sample cDNA was added to 18 μl reaction mix containing 10 μl of SYBR Green Realtime PCR Master Mix (Toyobo) and 8 μl of nuclease-free water (Sigma) with primers at a final concentration of 0.5 μM. All primer sequences are listed in **Supplementary Table 1**. The thermocycling was performed according to the manufacturer’s instructions. Experiments were performed in triplicate. The gene expression levels were calculated using the 2^−ΔCt^ method first normalizing to *GAPDH* expression as an endogenous control, and the expression level fold-change was calculated relative to the lowest expression level in the control group. 

### Cytotoxicity Assay

TP4 peptide (H-FIHHIIGGLFSAGKAIHRLIRRRRR-OH) was synthesized and purified by HPLC (GL Biochem), then diluted in sterile PBS before use. The preparation was sterilized by passing it through a syringe filter (PES membrane, pore size 0.22 μm, Millipore) to remove bacteria. The TP4 cytotoxicity was analyzed by MTS/PMS and lactate dehydrogenase (LDH) release assays. VK2 and 10% (v/v) GV sup-stimulated THP-1 and RAW264.7 cells were seeded on 96-well cell culture plates and treated with the indicated concentration of TP4 for 24 h. The positive control was 0.1% Triton-X 100. After incubation, the media were collected for the LDH release assay, and the wells were filled with 20 μl of MTS/PMS mixture (20:1) reagent (Promega) and 80 μl of cell growth medium, followed by incubation at 37°C for 1 h. Absorbance was recorded at a wavelength of 490 nm using a SpectraMax i3 Multi-Mode Microplate Reader. The collected cell culture supernatants were analyzed using a Cytotoxicity Detection Kit (LDH) (Roche) according to the manufacturer’s protocol. Briefly, 100 μl of supernatants were incubated with 100 μl LDH reaction mix for 10 min at room temperature. Then, 50 μl of stop solution was added before incubating the samples for 15 min at room temperature. The absorbance of the enzymatic product at 492 nm was measured using a SpectraMax i3 Multi-Mode Microplate Reader.

In the Annexin-V/PI staining assay, VK2 cells were suspended by trypsinization, washed twice with cold PBS, and incubated in binding buffer (Invitrogen) containing Annexin V-FITC (Invitrogen) and propidium iodide (PI) staining solution (Invitrogen) at room temperature for 15 min. Stained cells were analyzed by flow cytometry (Beckman Coulter, Indianapolis, IN, USA). The necrotic cells (C1), late apoptotic cells (C2), viable cells (C3), and early apoptotic cells (C4) were counted, and the percentage of apoptotic cells was determined as the sum of C2 and C4 divided by total cells × 100. The experiments were performed three times independently.

### Macrophage Treatments, Cell Co-Culture Model, and Wound−Healing Assay

In the direct and indirect TP4 treatment models (**Figure 3** and **Supplementary Figure 3**), PMA-differentiated THP-1 cells and RAW264.7 cells were stimulated with 10% (v/v) GV sup or control TSB broth in the serum-containing medium without antibiotics for 24 h. Then, the cells were washed and incubated in serum-free medium and treated with TP4 (7.82 µg/ml) or co-cultured with VK2 cells using cell culture inserts (pore size 0.4 μm; Corning). The insert-cultured VK2 cells had been pre-treated with vehicle (PBS) or 7.82 µg/ml TP4 for 8 h and then were changed to fresh KSFM. After 24 h incubation, THP-1 and RAW264.7 cells were collected, and the macrophage phenotypes were assessed.

To measure the anti-inflammatory and anti-apoptotic abilities of macrophages (**Figure 4**), VK2 cells were treated with 10% (v/v) GV sup or control TSB broth in KSFM without antibiotics. In co-culture groups, the VK2 cells were simultaneously co-cultured with THP-1/GV cells that had been pre-treated with TP4 (7.82 µg/ml) or vehicle (PBS) in complete medium for 24 h, followed by incubation in serum-free medium. After 24 h incubation, the cell culture media and VK2 cells were collected to perform measurements.

To investigate the wound repair ability of macrophages (**Figure 5**), a linear scratch was made on 100% confluent VK2 cell monolayers with a sterile pipette. The cells were then maintained in 10% (v/v) GV sup-containing KSFM and simultaneously co-cultured with THP-1/GV cells, which had been stimulated with vehicle (PBS)-treated or TP4-treated VK2 cells. The VK2 cells were subcultivated on an insert and incubated with serum-free medium after treatment. Wound healing was observed under a microscope (Leica) and analyzed by ImageJ software. Wound closure (%) = (A_0_ - A_n_)/A_0_ × 100, where A_0_ represents the area of initial wound area and A_n_ represents the remaining area of the wound at 24 h. After measurement, the cells were collected, and RNA was extracted.

### Detection of TNF-α, IL-1β, and IL-6 by MultiPlex Assay

The concentrations of cytokines TNF-α, IL-1β, and IL-6 in the VK2 cell culture supernatants were assessed by a MultiPlex assay (Bio-Rad). According to the manufacturer’s instructions, the cell culture supernatants were double centrifuged at 6,500 rpm for 15 min at 4°C to eliminate sediments. The samples were frozen at -80°C until being analyzed using a standard test system on a Bio-Plex 200 analyzer (Bio-Rad). Technical support was provided by the Academia Sinica Inflammation Core Facility, IBMS. Each experiment was performed in triplicate and concentrations were calculated based on a standard curve.

### Preparation of Conditioned Medium

To prepare the conditioned medium (CM), VK2 cells were seeded at more than 90% confluence and treated with TP4 (7.82 µg/ml) or vehicle (PBS) for 24 h. The next day, cells were washed with PBS, changed to fresh cell culture medium, and incubated for another day. The culture supernatants were then collected and centrifuged at 1,000 rpm for 10 min to remove any remaining cells or debris. For experiments that required neutralization of TNF-α-stimulated gene 6 (TSG-6) in the medium, 10 μg/ml of TSG-6 antibody (sc-398307; Santa Cruz Biotechnology) or control IgG (I9388; Sigma) was incubated with CM at room temperature for 1 h (36).

### Statistical Analysis

All data are expressed as mean ± SD. Statistical analyses were performed with GraphPad Prism Software. Multiple comparisons were performed by one-way or two-way analysis of variance (ANOVA). Significant intergroup differences were subsequently tested by *post hoc* Bonferroni analysis. *P* values of < 0.05 were considered statistically significant in all cases.

## Results

### M1 Polarization of THP-1 and RAW264.7 Cells After Treatment With *G. Vaginalis*-Free Culture Supernatants

**Figure 1:**
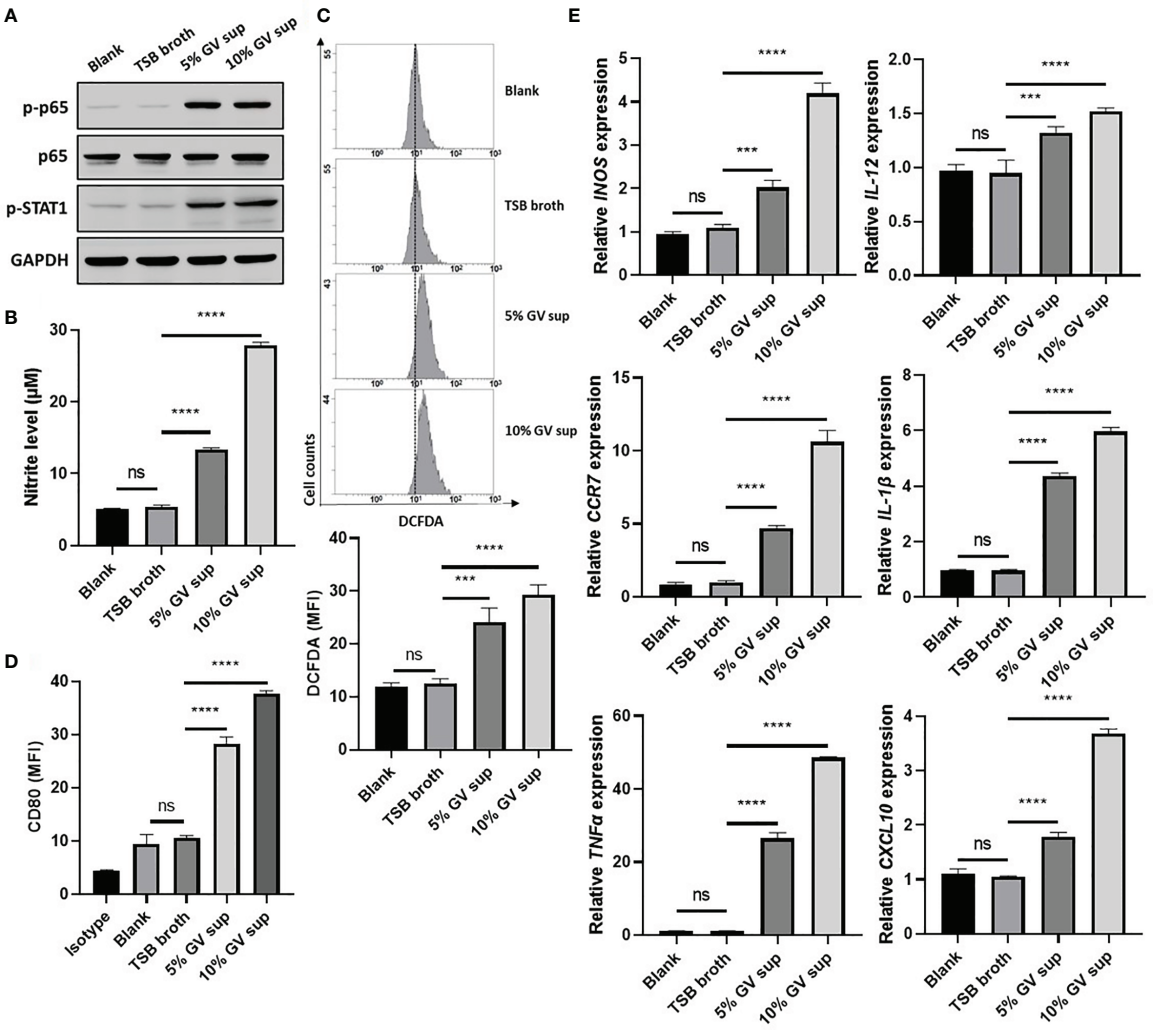
*G. vaginalis*-free culture supernatants (GV sup) induce M1 phenotype in THP-1 macrophages. PMA (phorbol 12-myristate 13-acetate)-differentiated THP-1 cells were stimulated with control medium (Blank), TSB broth, 5% or 10% (v/v) GV sup for 24 h. **(A)** Detection of phosphorylated STAT1 (p-STAT1) and NFκB p65 subunit (p-p65) by immunoblotting. GAPDH was used as an internal control to show equal protein loading. **(B)** Nitric oxide (NO) produced by the cells was assayed after treatment. **(C)** The reactive oxygen species (ROS) production was measured by DCFDA assay using flow cytometry. The mean fluorescence intensity (MFI) is shown in the bar graphs. **(D)** Macrophage surface marker (CD80) was detected by flow cytometry. The mean fluorescence intensity (MFI) is shown. The black bar graph indicates isotype controls. **(E)** Gene expression levels of M1 macrophage markers (*INOS, IL-12, CCR7, CXCL10, TNFα*, and *IL-1β*) were measured by qRT-PCR. Data were represented the normalized target gene amount relative to the blank group. Data are presented as mean ± SD of three independent experiments (***P < 0.001; ****P < 0.0001; ns, not statistically significant).

*G. vaginalis*-secreted virulence factors cause inflammatory responses and pro-inflammatory cytokine secretion in the female reproductive tract (9, 37). To determine if proinflammatory M1 macrophage could be induced by *G. vaginalis*-secreted factors, a human monocyte cell line (THP-1) and mouse macrophage cell line (RAW264.7) were incubated with 5% or 10% (v/v) *G. vaginalis*-free culture supernatants (GV sup) for 24 h. Immunoblot analysis revealed that compared to the control groups (blank and TSB broth), exposure of macrophages to GV sup increases the levels of phosphorylated nuclear factor-κB (NFκB) p65 (p-p65) and signal transducer and activator of transcription (STAT) 1 (p-STAT1) in both THP-1 and RAW264.7 cells (**Figure 1A** and **Supplementary Figure 1A**). Concurrently, the production of the microbicidal nitrite (**Figure 1B** and **Supplementary Figure 1B**) and reactive oxygen species (ROS) levels (**Figure 1C** and **Supplementary Figure 1C**) were enhanced after treatment. In addition, the elevation of M1-related surface marker CD80 (**Figure 1D** and **Supplementary Figure 1D**) and upregulated expression of pro-inflammatory genes (*INOS, IL-12, CCR7, CXCL10, TNFα*, and *IL-1β* in THP-1 cells, **Figure 1E**; *INOS*, *IL-12*, *CCR7*, *IL-6*, *TNFα*, and *IL-1β* in RAW264.7 cells, **Supplementary Figure 1E**) also indicated that *G. vaginalis*-secreted factors cause M1 macrophage polarization.

### Cytotoxicity of TP4 on Induced M1 Macrophages and Vaginal Epithelial Cell Lines

**Figure 2:**
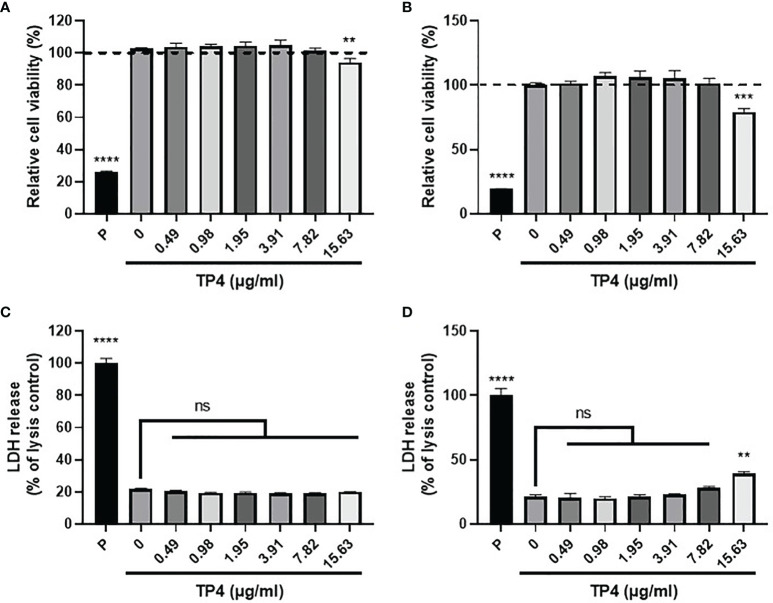
Cytotoxicity of TP4 in GV sup-induced THP-1 macrophages (THP-1/GV) and vaginal epithelial VK2 cells. THP-1/GV **(A)** and VK2 cells **(B)** were treated with different doses (0.49, 0.98, 1.95, 3.91, 7.82, 15.63 μg/ml) of TP4 for 24 h and subjected to MTS/PMS assay. Triton-X 100 (0.1%) served as a positive control (P). Results are shown as relative percentage to 0 μg/ml group. THP-1/GV **(C)** and VK2 **(D)** cells were treated with different doses (0.49, 0.98, 1.95, 3.91, 7.82, 15.63 μg/ml) of TP4 for 24 h. After treatment, the supernatants were subjected to an LDH release assay. Triton-X 100 (0.1%) served as a positive lysis control (P). Results are shown as relative percentage to positive control. Data are presented as mean ± SD of three independent experiments (**P < 0.01; ***P < 0.001; ****P < 0.0001; ns, not statistically significant, compared to 0 μg/ml).

Mucosal macrophage plasticity and polarization are not only controlled by autocrine mechanisms but also by microenvironmental factors, especially those secreted from epithelial cells (18, 38). To study the effects of TP4 treatment on macrophage polarization, we first established the non-toxic doses of TP4 on M1 macrophages induced from THP-1 and RAW264.7 cells (THP-1/GV and RAW264.7/GV) and the vaginal epithelial VK2 cells. MTS/PMS and lactate dehydrogenase (LDH) release assays were performed after treating cells with TP4 for 24 h. The MTS/PMS assay showed that TP4 treatment only slightly affected viability at a concentration of 15.63 µg/ml in THP-1/GV and VK2 cells (**Figures 2A, B**), and this dose had no cytotoxicity in RAW264.7/GV cells (**Supplementary Figure 2A**). In addition, TP4 treatment did not cause LDH release in THP-1/GV cells (**Figure 2C**), and it induced LDH release only at concentrations of at least 15.63 µg/ml in VK2 and RAW264.7/GV cells (**Figure 2D** and **Supplementary Figure 2B**). According to these results, we chose 7.82 μg/ml TP4 as the maximum concentration for the following experiments.

### TP4 Reprogramming of THP-1/GV and RAW264.7/GV Cells to M2 Phenotypes

**Figure 3:**
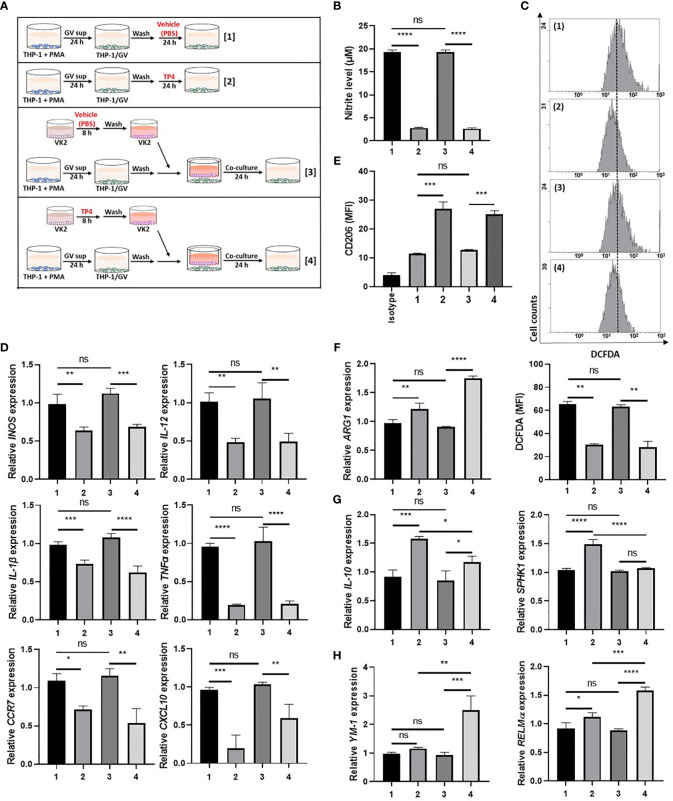
TP4 induces M2 phenotypes in THP-1/GV cells. **(A)** Schematic description of the TP4 treatment methods for THP-1/GV cells. PMA (phorbol 12-myristate 13-acetate)-differentiated THP-1 cells were stimulated with 10% (v/v) *G. vaginalis*-free culture supernatants (GV sup) for 24 h. After incubation, THP-1/GV cells were washed with PBS and treated with [treatment 1] vehicle (PBS) or [treatment 2] TP4 (7.82 µg/ml) for 24 h or co-cultured with VK2 cells which had been pre-treated with [treatment 3] vehicle (PBS) or [treatment 4] TP4 (7.82 µg/ml) on cell culture inserts for 8 h and then replaced with fresh culture medium. **(B)** The nitric oxide (NO) levels in THP-1/GV cells were assayed by the NO assay. **(C)** The reactive oxygen species (ROS) levels in THP-1/GV cells were measured by the DCFDA assay using flow cytometry. The mean fluorescence intensity (MFI) is shown in bar graphs. **(D)** Gene expression levels of M1-related genes (*INOS, IL-12, CCR7, CXCL10, TNFα*, and *IL-1β*) were measured by qRT-PCR. Data were represented the normalized target gene amount relative to the group of treatment 1. **(E)** Macrophage surface marker (CD206) was detected by flow cytometry; the MFI is shown. The black bar graph indicates isotype controls. **(F)** *ARG1* expression was measured by qRT-PCR. Data were represented the normalized target gene amount relative to the group of treatment 1. **(G)** Gene expression levels of M2c-related genes (*IL-10* and *SPHK1*) were measured by qRT-PCR. Data were represented the normalized target gene amount relative to the group of treatment 1. **(H)** Gene expression levels of M2a-related genes (*YM-1* and *RELMα*) were measured by qRT-PCR. Data were represented the normalized target gene amount relative to the group of treatment 1. Data are presented as mean ± SD of three independent experiments (*P < 0.05; **P < 0.01; ***P < 0.001; ****P < 0.0001; ns, not statistically significant).

Since GV sup strongly induced the M1 phenotype, we then used GV sup-induced macrophages to determine whether TP4 could affect the macrophage status. As shown in **Figure 3A** and **Supplementary Figure 3A**, we treated THP-1/GV and RAW264.7/GV cells with vehicle (PBS) [treatment 1] or TP4 (7.82 µg/ml) [treatment 2] for 24 h. Compared to the control cells (lane 1), nitrite production and intracellular ROS levels were significantly decreased after TP4 treatment (lane 2) (**Figures 3B, C** and **Supplementary Figures 3B, C**). Furthermore, expression levels of M1-related genes (*INOS, IL-12, CCR7, CXCL10, TNFα* and *IL-1β* in THP-1/GV cells; *INOS*, *IL-12*, *CCR7*, *IL-6*, *TNFα*, and *IL-1β* in RAW264.7/GV cells) were also reduced (**Figure 3D** and **Supplementary Figure 3D**), indicating that TP4 can decrease the inflammatory phenotypes of GV sup-induced macrophages.

Crosstalk between macrophages and epithelial cells may also affect macrophage status (38). Therefore, we used cell culture inserts to co-culture GV sup-induced macrophages with vaginal epithelial (VK2) cells, testing if TP4 treatment of vaginal epithelial cells might also affect macrophage status. As shown in **Figure 3A** and **Supplementary Figure 3A**, VK2 cells were seeded on cell culture inserts with small pores and treated with vehicle (PBS) [treatment 3] or TP4 (7.82 µg/ml) [treatment 4]. The treated VK2 cells were then co-cultured with THP-1/GV or RAW264.7/GV cells. Compared to the control co-culture group (lane 3), TP4-treatment of VK2 cells (lane 4) significantly decreased nitrite production, intracellular ROS levels, and M1-related gene expression (**Figures 3B–D** and **Supplementary Figures 3B-D**) in THP-1/GV and RAW264.7/GV cells. These results indicated that TP4 treatment of vaginal epithelial cells may also diminish the inflammatory phenotypes of M1 macrophages.

In addition to reducing inflammatory status, we found that M2 markers, CD206 and *arginase-1* (*ARG-1*), were simultaneously increased in both [treatment 2] and [treatment 4] samples (**Figures 3E, F** and **Supplementary Figures 3E, F**). However, no significant effects were observed when macrophages were treated with TP4 in the absence of GV sup stimulation (**Supplementary Figures 4A–E**). To further investigate which population of M2 macrophages, i.e., resolving (M2c) or wound-healing (M2a), were activated by TP4, we measured the expression levels of M2c-related genes (*IL-10* and *SPHK-1* in THP-1/GV cells; *IL-10* and *CD163* in RAW264.7/GV cells) and M2a-related genes (*YM-1* and *RELMα* in THP-1/GV cells; *YM-1* in RAW264.7/GV cells). Compared to control cells (lane 1), M2c-related genes were significantly increased after TP4 treatment (lane 2) in THP-1/GV and RAW264.7/GV cells (**Figure 3G** and **Supplementary Figure 3G**). Furthermore, compared to the co-culture controls (lane 3), M2a-related genes were significantly induced by co-culture with TP4-treated VK2 cells (lane 4) (**Figure 3H** and **Supplementary Figure 3H**). Together, these results indicated that TP4 can dampen the inflammatory phenotypes of GV sup-induced macrophages and can reprogram them either to the M2c type through direct effects or to the M2a type through effects on vaginal epithelial cells.

### Measurement of Tissue Remodeling Abilities in Different Types of TP4-Induced M2 Macrophages

Exposure to *G. vaginali*s induces inflammatory mediators and inhibits tissue repair in the vaginal epithelium, causing BV pathogenesis (37, 39). The actions of M2 macrophages include dampening inflammatory response (M2c type) and promoting tissue repair (M2a type), largely *via* secreted factors (18, 19). Since TP4 reprogrammed the status of inflammatory macrophages, we next evaluated the M2-related functions of TP4-treated macrophages.

**Figure 4:**
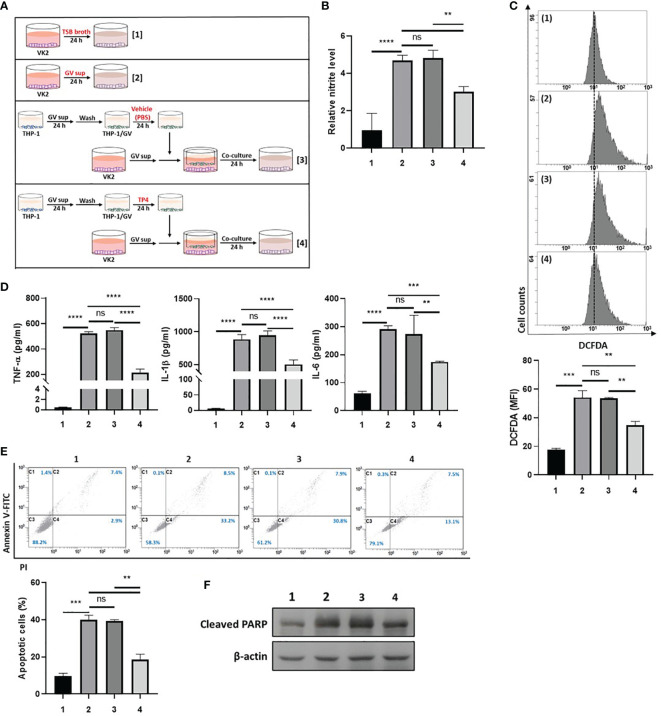
TP4-induced M2c macrophages attenuate GV sup-induced inflammation and cell death in VK2 cells. **(A)** Schematic description of the treatment methods. VK2 cells were treated with [treatment 1] TSB broth or [treatment 2] 10% (v/v) GV sup and co-cultured with THP-1/GV cells that had been pre-treated with [treatment 3] vehicle (PBS) or [treatment 4] TP4 (7.82 µg/ml) followed by incubation with serum-free medium. **(B)** The nitric oxide (NO) levels in VK2 cells were assayed by the NO assay. **(C)** The reactive oxygen species (ROS) levels in VK2 cells were measured by DCFDA assay using flow cytometry. The mean fluorescence intensity (MFI) is shown in the bar graphs. **(D)** The secretion of TNF-α (left), IL-1β (middle), and IL-6 (right) by the VK2 cells were measured by MultiPlex assay. **(E)** The treated cells were double-stained with PI and Annexin V and analyzed by flow cytometry. Four populations were identified: necrotic cells (C1), late apoptotic cells (C2), viable cells (C3), and early apoptotic cells (C4). Apoptotic cell percentage was quantified as the sum of C2 and C4 over total cell number × 100. Data are presented as mean ± SD of three independent experiments (**P < 0.01; ***P < 0.001; ****P < 0.0001; ns, not statistically significant). **(F)** Poly (ADP-ribose) polymerase (PARP) cleavage was detected by immunoblotting after co-culture. β-actin was used as an internal control to show equal protein loading.

First, we investigated the ability of TP4-induced M2c macrophages to prevent GV sup-induced epithelial inflammation and cell death. As shown in **Figure 4A**, VK2 cells were treated with control TSB broth [treatment 1] or 10% (v/v) GV sup [treatment 2] for 24 h to induce inflammation and cell death. Meanwhile, the GV sup-treated VK2 cells were co-cultured with THP-1/GV cells that had been previously treated with vehicle PBS [treatment 3] or TP4 [treatment 4] on the cell culture inserts. Compared to control broth (lane 1), GV sup (lane 2) significantly induced nitrite production (lane 2; **Figure 4B**), ROS levels (lane 2; **Figure 4C**), and secretion of inflammatory mediators (TNF-α, IL-1β, and IL-6) (lane 2; **Figure 4D**) in VK2 cells. Co-culture of GV sup-treated VK2 cells with TP4-treated THP-1/GV cells (lane 4) significantly decreased GV sup-induced inflammation (**Figures 4B–D**). Furthermore, Annexin-V/PI staining (**Figure 4E**) and the cleaved poly (ADPribose) polymerase (PARP) (**Figure 4F**) showed that exposure to GV sup caused apoptosis of VK2 cells, which could be attenuated by co-culture with TP4-treated THP-1/GV cells. These results indicated that TP4-induced M2c macrophages performed resolving functions to reduce inflammation and cell apoptosis.

**Figure 5:**
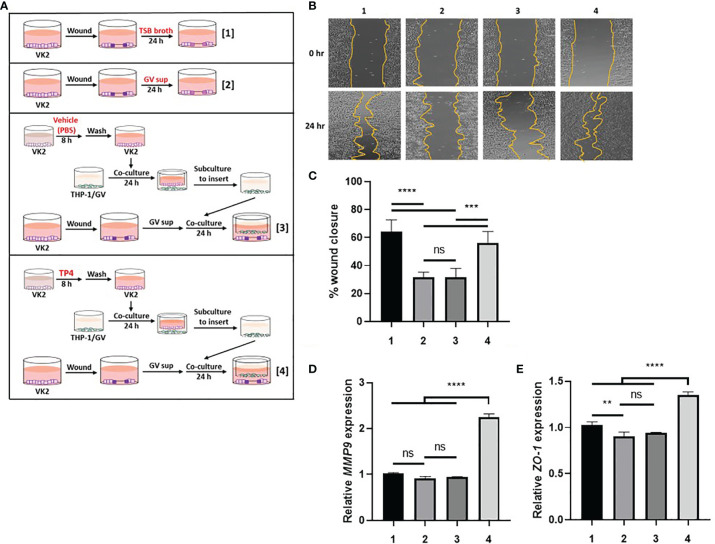
TP4-induced M2a macrophages promote VK2 cell migration and epithelial barrier integrity. **(A)** Schematic description of the treatment methods. VK2 cell monolayers were scratched with a sterile pipette and then treated with [treatment 1] TSB broth or [treatment 2] 10% (v/v) GV sup. Meanwhile, GV sup-treated VK2 cells were co-cultured with THP-1/GV cells which had been incubated with [treatment 3] vehicle (PBS)- or [treatment 4] TP4 (7.82 µg/ml)-treated VK2 cells. **(B)** Wound-healing assays were performed at 0 and 24 h in VK2 cells. **(C)** Wound closure was calculated as the percent area remaining uncovered by the cells at the given timepoint. **(D)** *MMP9* expression was measured by qRT-PCR after 24 h co-culture. **(E)** *ZO-1* expression was measured by qRT-PCR after 48 h co-culture. Data were represented the normalized target gene amount relative to the group of treatment 1. Data are presented as mean ± SD of three independent experiments (**P < 0.01; ***P < 0.001; ****P < 0.0001; ns, not statistically significant).

Second, we investigated if TP4-induced M2a macrophages could promote wound repair activities in GV sup-treated VK2 cells. As shown in **Figure 5A**, VK2 monolayers were scratched and treated with the control TSB broth [treatment 1] or 10% (v/v) GV sup [treatment 2]. Meanwhile, the GV sup-treated VK2 cells were co-cultured with THP-1/GV cells that had previously been stimulated with the vehicle PBS- [treatment 3] or TP4- [treatment 4] treated VK2 cells to induce a M2a phenotype. The results showed that treatment with GV sup decreased the wound closure ability of VK2 cells (lane 2; **Figures 5B, C**); however, co-culture with TP4-induced M2a macrophages accelerated the closure (lane 4; **Figures 5B, C**). Increased *matrix metalloproteinase-9* (*MMP-9*) expression was also detected (**Figure 5D**). Moreover, we found that expression of the tight junction component, *zonula occludens-1* (*ZO-1*), was enhanced after 48 h healing time (**Figure 5E**). These results indicated that TP4-induced M2a macrophages can not only promote epithelial cell migration but also increase epithelial barrier integrity after GV sup stimulation.

### TP4 Treatment Induces IL-10-STAT3 Activation *via* MAPK/ERK Pathway

**Figure 6:**
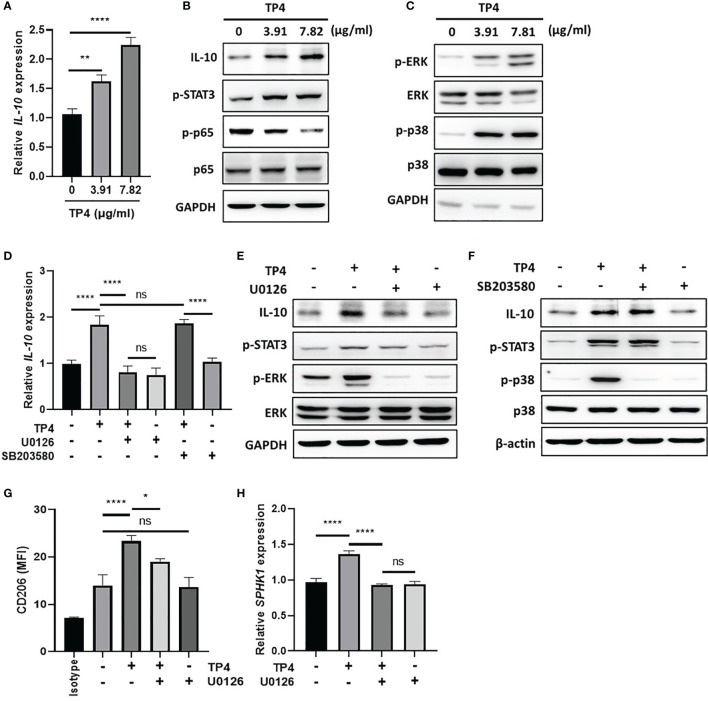
TP4 induces IL-10 expression *via* MAPK/ERK pathway in THP-1/GV cells and promotes M2c phenotypes. **(A)** *IL-10* expression was measured after 4 h TP4 (3.91 or 7.82 μg/ml) treatment of THP-1/GV cells by qRT-PCR. Data were represented the normalized target gene amount relative to the group of 0 μg/ml. **(B)** IL-10, and phosphorylated STAT3 (p-STAT3) and NFκB p65 subunit (p-p65) after 4 h TP4 (3.91 or 7.82 μg/ml) treatment of THP-1/GV cells were detected by immunoblotting. GAPDH was used as an internal control to show equal protein loading. **(C)** Detection of total and phosphorylated ERK (ERK/p-ERK) and p38 (p38/p-p38) after 4 h TP4 (3.91 or 7.82 μg/ml) treatment of THP-1/GV cells by immunoblotting. GAPDH was used as an internal control to show equal protein loading. **(D)** THP-1/GV cells were pre-treated with ERK inhibitor (U0126; 10 μM) or p38 inhibitor (SB203580; 10 μM) for 2 h in the serum-free medium. After incubation, TP4 (7.82 μg/ml) was treated for 4 h. *IL-10* expression was measured by qRT-PCR. Data were represented the normalized target gene amount relative to the untreated control group. **(E)** Detection of IL-10, p-STAT3, p-ERK, and ERK after U0126 (10 μM) and TP4 (7.82 μg/ml) treatments in THP-1/GV cells by immunoblotting. GAPDH was used as an internal control to show equal protein loading. **(F)** Detection of IL-10, p-STAT3, p-p38 and p38 after SB203580 (10 μM) and TP4 (7.82 μg/ml) treatments in THP-1/GV cells by immunoblotting. GAPDH was used as the internal control to show equal protein loading. **(G)** Macrophage surface marker (CD206) was detected by flow cytometry; the mean fluorescence intensity (MFI) is shown. The black bar graph indicates isotype controls. **(H)** *SPHK-1* expression after U0126 (10 μM) and TP4 (7.82 μg/ml) treatments in THP-1/GV cells were measured by qRT-PCR. Data were represented the normalized target gene amount relative to the untreated control group. Data are presented as mean ± SD of three independent experiments (*P < 0.05; **P < 0.01; ****P < 0.0001; ns, not statistically significant).

In response to inflammatory stimulation, IL-10 is rapidly expressed by macrophages through activation of the MAPK pathway, which results in suppression of NFκB signaling and activation of downstream STAT3 and M2c-related gene transcription (40, 41). Since TP4 is known to have a significant effect on MAPK signaling (42), and we found *IL-10* was strongly induced upon TP4 treatment in macrophages (**Figure 3G** and **Supplementary Figure 3G**), we hypothesized that TP4 may regulate IL-10 expression through activation of the MAPK pathway. Using qRT-PCR and immunoblotting, we observed that mRNA (**Figure 6A** and **Supplementary Figure 5A**) and protein (**Figure 6B** and **Supplementary Figure 5B**) levels of IL-10 were dose-dependently increased by TP4 treatment, and this observation corresponded to increased STAT3 phosphorylation (p-STAT3) and decreased p-p65 in THP-1/GV and RAW264.7/GV cells. Although the active forms of ERK (p-ERK) and p38 (p-p38) were increased after TP4 treatment (**Figure 6C** and **Supplementary Figure 5C**), only pre-treatment with an ERK pathway inhibitor, U0126, could suppress the TP4-induced IL-10 expression and p-STAT3 (**Figures 6D–F** and **Supplementary Figures 5D–F**). Further, inhibition of the ERK pathway also repressed the TP4-induced expression of M2 marker CD206 (**Figure 6G** and **Supplementary Figure 5G**), as well as *SPHK1* in THP-1/GV cells (**Figure 6H**), and *CD163* in RAW264.7/GV cells (**Supplementary Figure 5H**). These results demonstrated that treatment of TP4 induces IL-10 expression through the MAPK/ERK pathway, resulting in activation of downstream STAT3 and M2c-related genes.

### TP4-Induced TNF-α-Stimulated Gene 6 (TSG-6) Secretion From VK2 Cells Is Required for STAT6 Activation and M2a Macrophage Polarization

**Figure 7:**
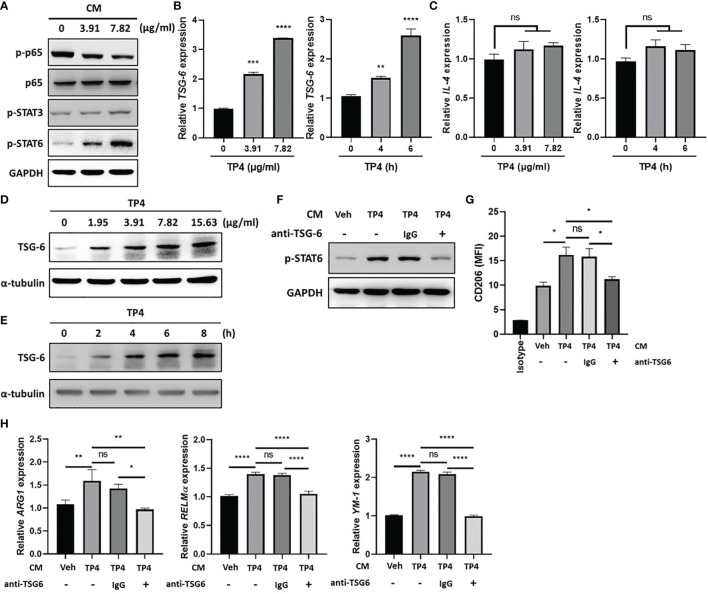
TP4-induced TNF-α-stimulated gene 6 (TSG-6) secretion from VK2 cells is required for STAT6 activation in THP-1/GV cells. **(A)** Detection of phosphorylated NFκB p65 subunit (p-p65), STAT3 (p-STAT3), and STAT6 (p-STAT6) after 1 h treatment with conditioned medium (CM) from TP4 (3.91 or 7.82 μg/ml)-treated VK2 cells to THP-1/GV cells by immunoblotting. GAPDH was used as an internal control to show equal protein loading. **(B)** *TSG-6* expression in VK2 cells after TP4 (3.91 or 7.82 μg/ml) treatment for 6 h (left) or 7.82 μg/ml TP4 treatments for 0, 4 or 6 h (right), measured by qRT-PCR. Data were represented the normalized target gene amount relative to the group of 0 μg/ml. **(C)** *IL-4* expression in VK2 cells after TP4 (3.91 or 7.82 μg/ml) treatment for 6 h (left) or 7.82 μg/ml TP4 treatments for 0, 4 or 6 h (right), measured by qRT-PCR. Data were represented the normalized target gene amount relative to the group of 0 μg/ml. **(D)** Detection of TSG-6 expression in VK2 cells after 6 h treatment of TP4 (1.95, 3.91, 7.82 or 15.63 μg/ml) by immunoblotting. α-tubulin was used as an internal control to show equal protein loading. **(E)** Detection of TSG-6 expression in VK2 cells after 7.82 μg/ml TP4 treatment for 0, 2, 4, 6, or 8 h by immunoblotting. α-tubulin was used as an internal control to show equal protein loading. **(F)** The CM from vehicle (Veh; PBS)- or TP4-treated VK2 cells were incubated with TSG-6-neutralizing antibody (anti-TSG6) or IgG control for 1 h and then used to treat THP-1/GV cells. After incubation, p-STAT6 in THP-1/GV cells was detected. GAPDH was used as an internal control to show equal protein loading. **(G)** Macrophage surface marker (CD206) was detected by flow cytometry after CM incubation. The mean fluorescence intensity (MFI) is shown. The black bar graph indicates isotype controls. **(H)** Gene expression levels of *ARG1, YM-1*, and *RELMα* were measured after treatments by qRT-PCR. Data were represented the normalized target gene amount relative to the vehicle-treated group (lane 1). Data are presented as mean ± SD of three independent experiments (*P < 0.05; **P < 0.01; ***P < 0.001; ****P < 0.0001; ns, not statistically significant).

Using the co-culture model shown in **Figure 3**, we found that TP4 treatment of insert-cultured VK2 cells promoted the M2a phenotype. This finding suggested that identifiable determinants of M2a polarization may be produced by VK2 cells after TP4 treatment. To investigate the underlying mechanisms of this effect, we collected the conditioned medium (CM) from TP4-treated VK2 cells and used it to treat THP-1/GV and RAW264.7/GV cells. After treatment, p-p65 was decreased, and compared to p-STAT3, the phosphorylation of STAT6 (p-STAT6) was strongly increased in THP-1/GV (**Figure 7A**) and RAW264.7/GV cells (**Supplementary Figure 7A**). STAT6 plays a pivotal role in M2a macrophage differentiation and can be stimulated by soluble factors, such as IL-4 and TNF-α-stimulated gene 6 (TSG6) (21, 43). Indeed, we found that TSG-6 was upregulated by TP4 not only in VK2 (**Figures 7B, D, E**) but also in endocervical epithelial (End1) cells in dose- and time-dependent manners (**Supplementary Figures 6A, C, D**). However, IL-4 was not changed (**Figure 7C** and **Supplementary Figure 6B**), indicating that TP4 may modulate macrophage status through TSG-6 secretion by vaginal epithelial cells. To test this hypothesis, TSG-6-neutralizing antibodies were added to the CM from TP4-treated VK2 cells. We found that the addition of TSG-6-neutralizing antibody reduced the increased STAT6 phosphorylation (**Figure 7F** and **Supplementary Figure 7B**), CD206 upregulation (**Figure 7G** and **Supplementary Figure 7C**), and M2a-related gene expression (*ARG-1*, *YM-1*, and *RELMα* in THP-1/GV cells; *ARG-1* and *YM-1* in RAW264.7/GV cells) (**Figure 7H** and **Supplementary Figure 7D**). These results confirmed that TP4-induced TSG-6 secretion from epithelial cells activated STAT6 signaling in the macrophages and reprogrammed them to the M2a phenotype.

## Discussion

BV is a vaginal inflammatory disease caused by dysregulation of commensal bacteria. It is difficult to treat and has high rates of recurrence (44). Metronidazole is the current treatment option for BV, as it can kill *G. vaginalis* (45). However, almost 60% of *G. vaginalis* isolates taken from patients are resistant to metronidazole (6). In addition, metronidazole exhibits an unwanted suppressive effect on vaginal *Lactobacilli* (46). Thus, antibiotics seem to be a suboptimal treatment option for BV, due to their non-selective killing effect on vaginal flora. Instead, modulation of inflammatory status and epithelial barrier function in the vagina have been proposed as alternative therapeutic strategies (47, 48). Our study demonstrated that TP4 has multiple beneficial effects in the context of BV. As such, TP4 can promote M2 macrophage polarization, especially M2c and M2a (**Figures 3**, **4**). In addition, TP4 can promote tissue remodeling processes of the vaginal epithelium (**Figure 5**). Both of these effects are beneficial for relieving BV.

TP4 is able to reprogram pro-inflammatory M1 macrophages into tissue repair M2a macrophages and resolving M2c macrophages (**Figure 3**). This reprogramming seems to occur *via* three possible mechanisms. First, TP4 stimulates macrophage phenotype switching *via* induction of IL-10 (**Figure 6** and **Supplementary Figure 5**), which is a key cytokine for promoting M2c macrophage polarization (49). Second, TP4 induces TSG-6 in epithelial cells. VK2 vaginal epithelial (**Figure 7**) and End1 endocervical epithelial (**Supplementary Figure 6**) cells exposed to TP4 showed upregulation of TSG-6, which activated STAT6 in macrophages. TSG-6 has been considered as a therapeutic target in many inflammation-related diseases, including atherosclerosis (50), osteoarthritis (51), acute myocardial infarction (52), subarachnoid hemorrhage-induced early brain injury (53), inflammatory lung injury (54), and alcoholic hepatitis (55). In addition to its anti-inflammatory activity, TSG-6 also exhibits tissue-protective activity (56). It is able to alleviate tissue injury and enhance the healing process (56). This protective activity of TSG-6 is consistent with our findings, which showed that TSG-6 mediates TP4-induced M2a macrophage polarization (**Figure 7** and **Supplementary Figure 7**). M2a macrophages play an important role in anti-inflammation and tissue repair through induction of IL-10 (41) and regulation of tissue remodeling (57), respectively. The third mechanism may be neutralization of soluble pathogen-associated molecular patterns (PAMPs) or toxins released from *G. vaginalis*. There are several molecules released from *G. vaginalis* that are associated with BV, including phospholipases, cholesterol-dependent cytolysin, and vaginolysin (58, 59). Vaginolysin is considered to be a major cause of vaginal epithelial cytotoxicity and induction of inflammatory response (59, 60). Notably, TP4 is a cationic antimicrobial peptide (29), and cationic antimicrobial peptides are able to suppress negatively charged PAMP-induced inflammation *via* electrostatic attraction-mediated neutralization activity (61). However, the ability of TP4 to directly neutralize *G. vaginalis*-related PAMPs has not been demonstrated.

Maintaining the integrity of the vaginal epithelial barrier is essential for preventing sexually transmitted diseases caused by harmful microorganisms (62). Patients with BV have increased susceptibility to other sexually transmitted disease pathogens, such as HIV (63). This increased susceptibility is due to immune activation (64), recruitment of HIV target cells (65), and damage to vaginal epithelial barriers (66). Our findings suggest that TP4 might improve vaginal barrier integrity *via* suppression of epithelial cell inflammation and apoptosis (**Figure 4**) and promotion of epithelial cell migration (**Figure 5**). M2 macrophages suppress apoptosis *via* various mechanisms, such as inhibition of proapoptotic mechanisms (67) and induction of IL-10 secretion (41). IL-10 has anti-apoptotic effects on various types of cells (68–70). Therefore, TP4 might reduce the risk of BV patients contracting other sexually transmitted diseases. Furthermore, TP4 might also facilitate the rebuilding of a healthy vaginal microenvironment and reduce the recovery period of BV. The vaginal epithelium is more than a physical barrier, as it plays a critical role in the regulation of vaginal immunity (71). Impairments in vaginal epithelium function are associated with higher inflammatory status driven by M1 macrophage accumulation (72). The M1 macrophage accumulation usually prolongs the inflammatory phase and increases disease severity *via* cytokine production (73).

TP4 elevates IL-10 expression in macrophages (**Figure 6** and **Supplementary Figure 5**), which might provide clinical benefits in BV. IL-10 is a key anti-inflammatory cytokine that is essential for ending the inflammatory phase by promoting macrophage polarization toward anti-inflammatory phenotypes (49). Furthermore, elevated expression of IL-10 is correlated with improved probiotic colonization in the vagina (74). In contrast, low levels of IL-10 in BV patients are associated with increased risk of adverse pregnancy outcomes (74).

In addition to *G. vaginalis* (58, 59), several other pathogens have been implicated in BV (75). TP4 possesses demonstrated killing activity toward several vaginosis-related pathogens, including *Candida albicans*, *Staphylococcus aureus*, and *Escherichia coli* (29). However, whether TP4 has a direct killing effect on *G. vaginalis* remains unclear.

**Figure 8:**
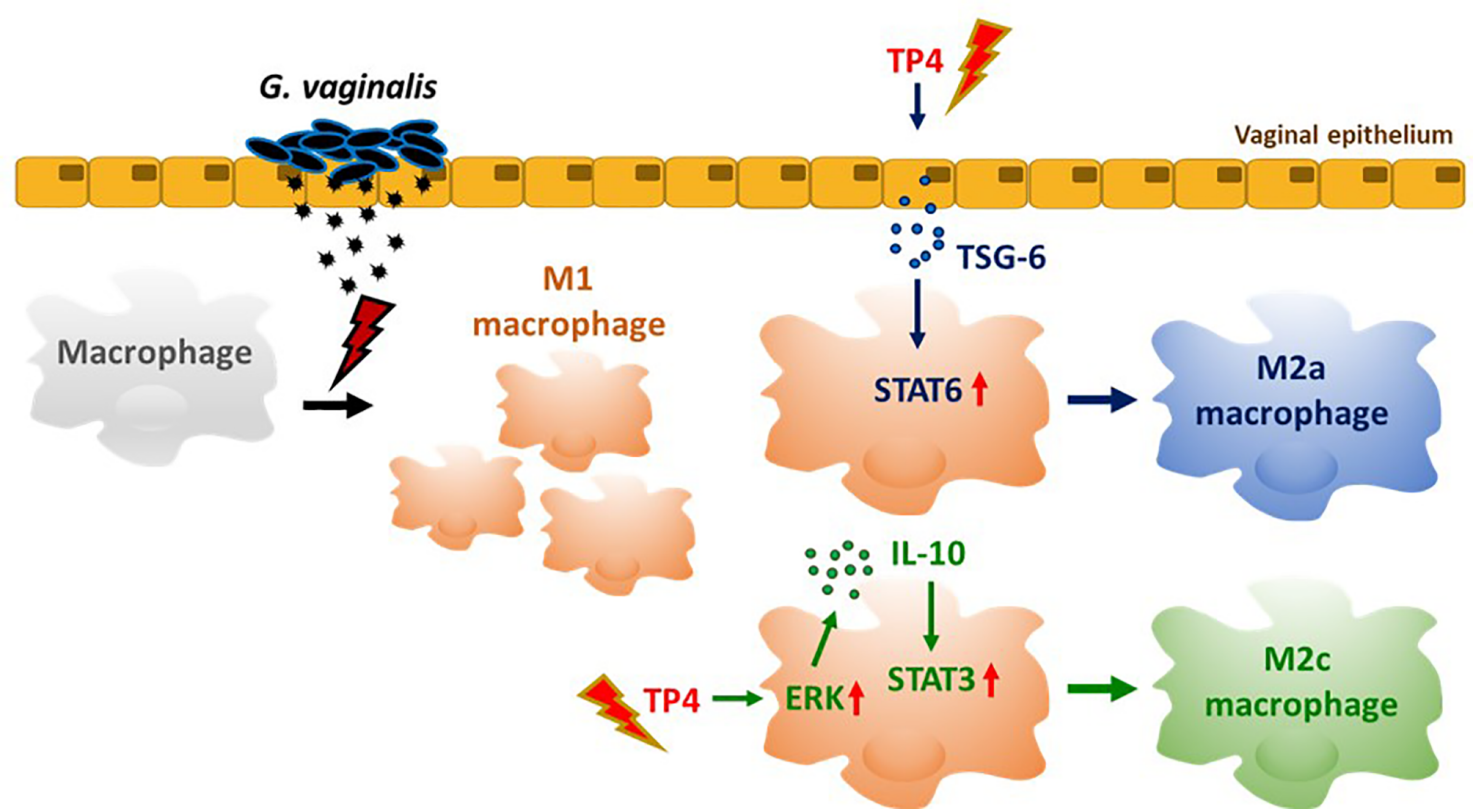
TP4-mediated macrophage reprogramming. *G. vaginalis*-secreted virulence factors cause macrophages to exhibit a pro-inflammatory M1 type polarization. Treatment with TP4 switches the M1 macrophages toward resolving (M2c) or tissue repair (M2a) phenotypes. Direct application of TP4 to macrophages induces IL-10 expression *via* the MAPK/ERK pathway, which results in activation of downstream STAT3 signaling and promotion of M2c-related phenotypes. On the other hand, TP4 promotes vaginal epithelial cells to secret TSG-6, which in turn induces activation of downstream STAT6 signaling and promotes M2a-related phenotypes.

In summary (**Figure 8**), TP4 exhibits several anti-inflammatory properties (**Figure 3**). TP4 elevates IL-10 in macrophages, which is followed by M2c macrophage polarization (**Figure 6**). It also induces M2a macrophage polarization *via* induction of TSG-6 in vaginal epithelial cells (**Figure 7**). Moreover, the virulence of *G*. *vaginalis* could potentially be mitigated by TP4-induced M2c and M2a macrophages *via* suppression of vaginal epithelial cell inflammation and apoptosis (**Figure 4**) and induction of vaginal epithelial cell migration (**Figure 5**), respectively. Since we successfully identified the immunomodulatory activities of TP4 in the vaginal microenvironment using an *in vitro* model, further investigations using primary cells and *in vivo* models are warranted to verify the pharmacological potential of TP4 in BV.

## Data Availability Statement

The original contributions presented in the study are included in the article/**Supplementary Material**. Further inquiries can be directed to the corresponding author.

## Author Contributions

C-WL and B-CS wrote the paper. J-YC supervised the study and edited the paper. C-WL studied the experiments. All authors contributed to the article and approved the submitted version.

## Funding

All research funds were obtained from the Marine Research Station, Institute of Cellular and Organismic Biology, Academia Sinica, Jiaushi, Ilan, Taiwan to J-YC. J-YC received a grant from the Ministry of Science and Technology, Taiwan (MOST 109-2811-B-001-562). This work was supported in part by the Ministry of Science and Technology (110-2622-B-001 -002 -)(108-2313-B-001 -006 -) in Taiwan.

## Conflict of Interest

The authors declare that the research was conducted in the absence of any commercial or financial relationships that could be construed as a potential conflict of interest.

## Publisher’s Note

All claims expressed in this article are solely those of the authors and do not necessarily represent those of their affiliated organizations, or those of the publisher, the editors and the reviewers. Any product that may be evaluated in this article, or claim that may be made by its manufacturer, is not guaranteed or endorsed by the publisher.

## Supplementary Material

Supplementary Figure 1
*G. vaginalis*-free culture supernatants (GV sup) induce M1 phenotype in RAW264.7 macrophages. RAW264.7 cells were stimulated with control medium (Blank), TSB broth, 5% or 10% (v/v) GV sup for 24 h. **(A)** Detection of phosphorylated STAT1 (p-STAT1) and NFκB p65 subunit (p-p65) by immunoblotting. α-Tubulin was used as an internal control to show equal protein loading. **(B)** The nitric oxide (NO) produced by the cells was assayed after treatment. **(C)** The reactive oxygen species (ROS) production was measured by DCFDA assay using flow cytometry. The mean fluorescence intensity (MFI) is shown in bar graphs. **(D)** Macrophage surface marker (CD80) was detected by flow cytometry. The mean fluorescence intensity (MFI) is indicated. The black bar graph indicates isotype controls. **(E)** Gene expression levels of M1 macrophage markers (*INOS, IL-12, CCR7, IL-6, TNFα*, and *IL-1β*) were measured by qRT-PCR. Data were represented the normalized target gene amount relative to the blank group. Data are presented as mean ± SD of three independent experiments (*, *P* < 0.05; **, *P* < 0.01; ***, *P* < 0.001; ****, *P* < 0.0001; ns, not statistically significant).

Supplementary Figure 2 Cytotoxicity of TP4 on GV sup-induced RAW264.7 macrophages (RAW264.7/GV). **(A)** RAW264.7/GV cells were treated with different doses (0.49, 0.98, 1.95, 3.91, 7.82, 15.63 μg/ml) of TP4 for 24 h and subjected to MTS/PMS assay. Triton-X 100 (0.1%) served as a positive control (P). Results are shown as relative percentage to 0 μg/ml group. **(B)** RAW264.7/GV cells were treated with different doses (0.49, 0.98, 1.95, 3.91, 7.82, 15.63 μg/ml) of TP4 for 24 h. After treatment, the supernatants were subjected to an LDH release assay. Triton-X 100 (0.1%) served as a positive lysis control (P). Results are indicated as a relative percentage to positive lysis control. Data are presented as mean ± SD of three independent experiments (*, *P* < 0.05; **, *P* < 0.01; ***, *P* < 0.001; ****, *P* < 0.0001; ns, not statistically significant, compared to 0 μg/ml).

Supplementary Figure 3 TP4 induces M2 phenotypes in RAW264.7/GV cells. **(A)** Schematic description of the TP4 treatment methods for RAW264.7/GV cells. RAW264.7 cells were stimulated with 10% (v/v) *G. vaginalis*-free culture supernatants (GV sup) for 24 h. After incubation, RAW264.7/GV cells were washed with PBS and treated with [treatment 1] vehicle (PBS) or [treatment 2] TP4 (7.82 µg/ml) for 24 h or co-cultured with VK2 cells which had been pre-treated with [treatment 3] vehicle (PBS) or [treatment 4] TP4 (7.82 µg/ml) for 8 h and then replaced with fresh culture medium on cell culture inserts. **(B)** The nitric oxide (NO) levels in RAW264.7/GV cells were assayed by the NO assay. **(C)** The reactive oxygen species (ROS) levels in RAW264.7/GV cells were measured by DCFDA assay using flow cytometry. The mean fluorescence intensity (MFI) is shown in the bar graphs. **(D)** Gene expression levels of M1-related genes (*INOS, IL-12, CCR7, TNFα*, and *IL-1β*) were measured by qRT-PCR. Data were represented the normalized target gene amount relative to the group of treatment 1. **(E)** Macrophage surface marker (CD206) was detected by flow cytometry; the MFI is shown. The black bar graph indicates isotype controls. **(F)** *ARG1* expression was measured by qRT-PCR. Data were represented the normalized target gene amount relative to the group of treatment 1. **(G)** *IL-10* and *CD163* expression levels were measured by qRT-PCR. Data were represented the normalized target gene amount relative to the group of treatment 1. **(H)** *YM-1* expression was measured by qRT-PCR. Data were represented the normalized target gene amount relative to the group of treatment 1. Data are presented as mean ± SD of three independent experiments (*, *P* < 0.05; **, *P* < 0.01; ***, *P* < 0.001; ****, *P* < 0.0001; ns, not statistically significant).

Supplementary Figure 4 TP4 does not affect M1/M2 markers in the absence of GV sup stimulation. PMA-differentiated THP-1 and RAW264.7 cells were treated with vehicle PBS (Veh) or TP4 (7.82 µg/ml) for 24 h. A macrophage surface marker (CD206) was detected by flow cytometry in THP-1 **(A)** and RAW264.7 **(B)** cells; the MFI is shown. The black bar graph indicates isotype controls. *ARG1* expression levels in THP-1 **(C)** and RAW264.7 **(D)** cells were measured by qRT-PCR. Data represent the normalized target gene relative to the vehicle group. Nitric oxide produced by THP-1 **(E)** and RAW264.7 **(F)** cells was assayed after TP4 treatment. Data are presented as mean ± SD of three independent experiments (ns, not statistically significant).

Supplementary Figure 5 TP4 induces IL-10 expression *via* MAPK/ERK pathway in RAW264.7/GV cells and promotes M2c phenotypes. **(A)** *IL-10* expression after 4 h TP4 (3.91 or 7.82 μg/ml) treatment of RAW264.7/GV cells was measured by qRT-PCR. Data were represented the normalized target gene amount relative to the group of 0 μg/ml. **(B)** IL-10, and phosphorylated STAT3 (p-STAT3) and NFκB p65 subunit (p-p65) after 4 h TP4 (3.91, 7.82 μg/ml) treatment of RAW264.7/GV cells were detected by immunoblotting. GAPDH was used as an internal control to show equal protein loading. **(C)** Detection of total and phosphorylated ERK (ERK/p-ERK) and p38 (p38/p-p38) after 4 h TP4 (3.91 or 7.82 μg/ml) treatment of RAW264.7/GV cells by immunoblotting. GAPDH was used as an internal control to show equal protein loading. **(D)** RAW264.7/GV cells were pre-treated with ERK inhibitor (U0126; 10 μM) or p38 inhibitor (SB203580; 10 μM) for 2 h in serum-free medium. After incubation, TP4 (7.82 μg/ml) was treated for 4 h. *IL-10* expression after treatments was measured by qRT-PCR. Data were represented the normalized target gene amount relative to the untreated control group. **(E)** Detection of IL-10, p-STAT3, p-ERK, and ERK after U0126 (10 μM) and TP4 (7.82 μg/ml) treatments for 4 h in RAW264.7/GV cells by immunoblotting. GAPDH was used as an internal control to show equal protein loading. **(F)** Detection of IL-10, p-STAT3, p-p38, and p38 after SB203580 (10 μM) and TP4 (7.82 μg/ml) treatments for 4 h in RAW264.7/GV cells by immunoblotting. β-actin was used as an internal control to show equal protein loading. **(G)** Macrophage surface marker (CD206) was detected by flow cytometry; the mean fluorescence intensity (MFI) is shown. The black bar graph indicates isotype controls. **(H)** *CD163* expression after U0126 (10 μM) and TP4 (7.82 μg/ml) treatments in RAW264.7/GV cells were measured by qRT-PCR. Data were represented the normalized target gene amount relative to the untreated control group. Data are presented as mean ± SD of three independent experiments (*, *P* < 0.05; **, *P* < 0.01; ***, *P* < 0.001; ns, not statistically significant).

Supplementary Figure 6 TP4 induces TNF-α-stimulated gene 6 (TSG-6) secretion from the endocervical epithelial (End1) cells. **(A)** *TSG-6* expression after TP4 (3.91 or 7.82 μg/ml) treatments for 6 h (left) and 7.82 μg/ml TP4 treatments for 0, 4 or 6 h (right) in End1 cells, measured by qRT-PCR. Data were represented the normalized target gene amount relative to the group of 0 μg/ml. **(B)** *IL-4* expression after TP4 (3.91 or 7.82 μg/ml) treatments for 6 h (left) and 7.82 μg/ml TP4 treatment for 0, 4 or 6 h (right) in End1 cells, measured by qRT-PCR. Data were represented the normalized target gene amount relative to the group of 0 μg/ml. Data are presented as mean ± SD of three independent experiments (*, *P* < 0.05; **, *P* < 0.01; ***, *P* < 0.001; ****, *P* < 0.0001; ns, not statistically significant, compared to 0 μg/ml). **(C)** Detection of TSG-6 expression in End1 cells after treatment of TP4 (1.95, 3.91, 7.82 or 15.63 μg/ml) for 6 h by immunoblotting. α-tubulin was used as an internal control to show equal protein loading. **(D)** Detection of TSG-6 expression in End1 cells after 7.82 μg/ml TP4 treatment for 0, 2, 4, 6 or 8 h by immunoblotting. α-tubulin was used as an internal control to show equal protein loading.

Supplementary Figure 7 TP4-induced TNF-α-stimulated gene 6 (TSG-6) secretion from VK2 cells is required for STAT6 activation in RAW264.7/GV cells. **(A)** Detection of phosphorylated NFκB p65 subunit (p-p65), STAT3 (p-STAT3), and STAT6 (p-STAT6) after 1 h treatment with the conditioned medium (CM) from TP4 (3.91 or 7.82 μg/ml)-treated VK2 cells to RAW264.7/GV cells by immunoblotting. β-actin was used as an internal control to show equal protein loading. **(B)** The CM from vehicle (Veh; PBS)- or TP4-treated VK2 cells were incubated with TSG-6 neutralizing antibody (anti-TSG6) or IgG control for 1 h and then used to treat RAW264.7/GV cells. After incubation, p-STAT6 in RAW264.7/GV cells was detected. GAPDH was used as an internal control to show equal protein loading. **(C)** Macrophage surface marker (CD206) was detected by flow cytometry after CM treatments. The mean fluorescence intensity (MFI) is shown. The black bar graph indicates isotype controls. **(D)** *ARG1* (left) and *YM-1* (right) expression levels were measured after treatments by qRT-PCR. Data were represented the normalized target gene amount relative to the vehicle-treated group (lane 1). Data are presented as mean ± SD of three independent experiments (*, *P* < 0.05; **, *P* < 0.01; ***, *P* < 0.001; ****, *P* < 0.0001; ns, not statistically significant).
