# Supplementary figures and images for: Tilapia Piscidin 4 (TP4) Reprograms M1 Macrophages to M2 Phenotypes in Cell Models of Gardnerella vaginalis-Induced Vaginosis
Source: Front Immunol. 2021 Dec 2;12:773013. doi: 10.3389/fimmu.2021.773013 (PMC8674419; doi:10.3389/fimmu.2021.773013)

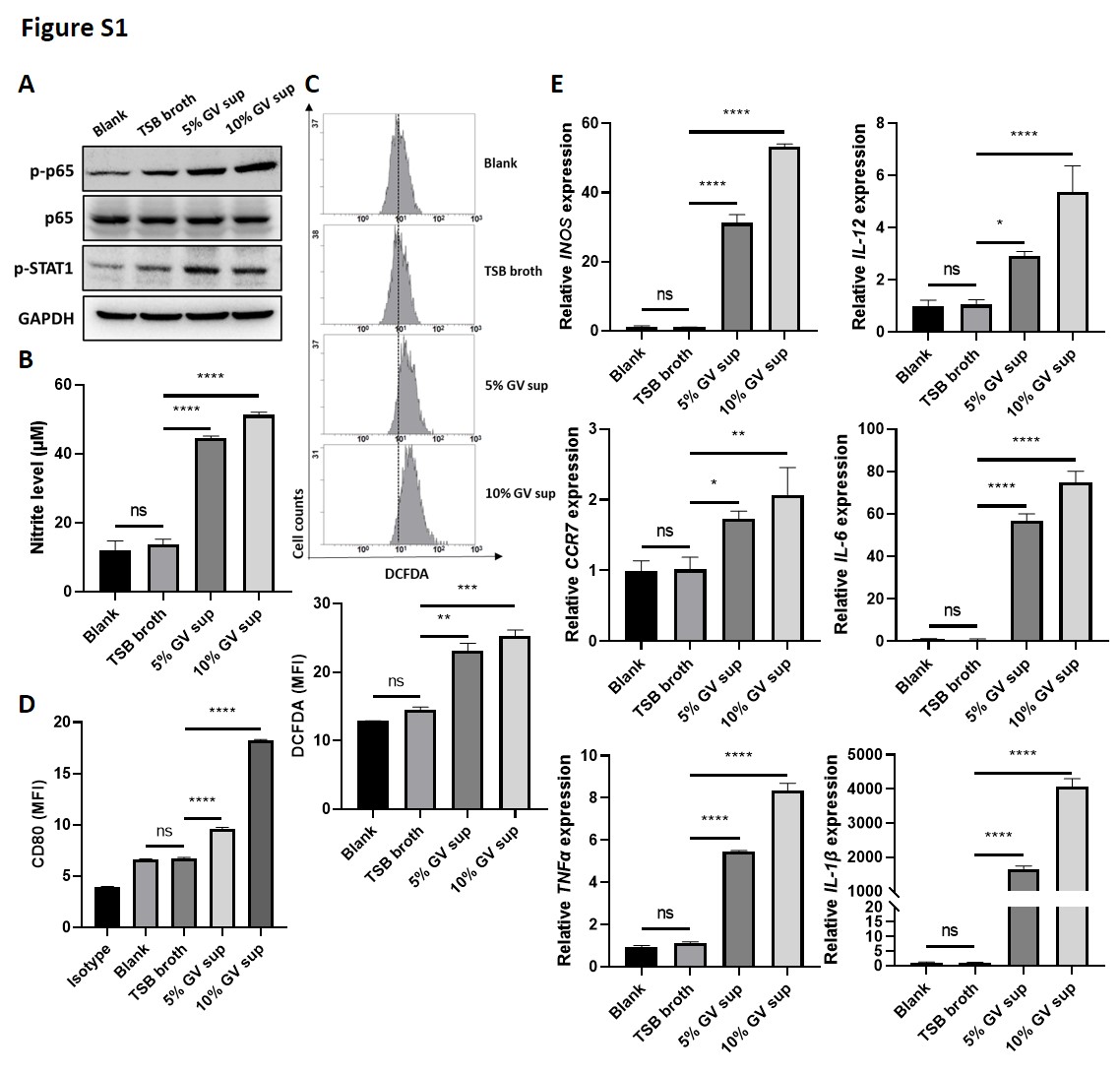

Supplement: Supplementary Figure 1 — G. vaginalis-free culture supernatants (GV sup) induce M1 phenotype in RAW264.7 macrophages. RAW264.7 cells were stimulated with control medium (Blank), TSB broth, 5% or 10% (v/v) GV sup for 24 h. (A) Detection of phosphorylated STAT1 (p-STAT1) and NFκB p65 subunit (p-p65) by immunoblotting. α-Tubulin was used as an internal control to show equal protein loading. (B) The nitric oxide (NO) produced by the cells was assayed after treatment. (C) The reactive oxygen species (ROS) production was measured by DCFDA assay using flow cytometry. The mean fluorescence intensity (MFI) is shown in bar graphs. (D) Macrophage surface marker (CD80) was detected by flow cytometry. The mean fluorescence intensity (MFI) is indicated. The black bar graph indicates isotype controls. (E) Gene expression levels of M1 macrophage markers (INOS, IL-12, CCR7, IL-6, TNFα, and IL-1β) were measured by qRT-PCR. Data were represented the normalized target gene amount relative to the blank group. Data are presented as mean ± SD of three independent experiments (*, P < 0.05; **, P < 0.01; ***, P < 0.001; ****, P < 0.0001; ns, not statistically significant). [file Image_1.jpeg]

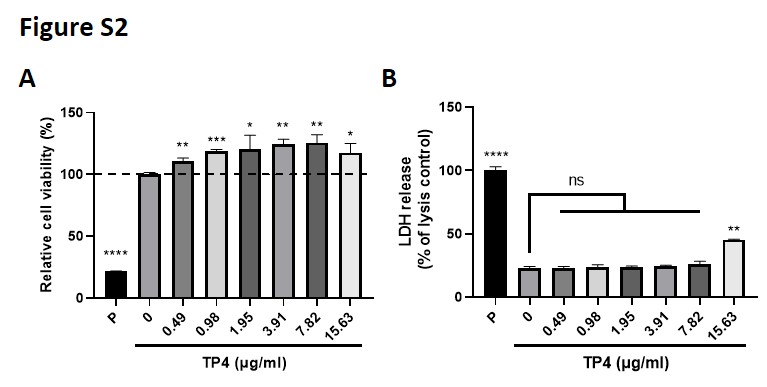

Supplement: Supplementary Figure 2 — Cytotoxicity of TP4 on GV sup-induced RAW264.7 macrophages (RAW264.7/GV). (A) RAW264.7/GV cells were treated with different doses (0.49, 0.98, 1.95, 3.91, 7.82, 15.63 μg/ml) of TP4 for 24 h and subjected to MTS/PMS assay. Triton-X 100 (0.1%) served as a positive control (P). Results are shown as relative percentage to 0 μg/ml group. (B) RAW264.7/GV cells were treated with different doses (0.49, 0.98, 1.95, 3.91, 7.82, 15.63 μg/ml) of TP4 for 24 h. After treatment, the supernatants were subjected to an LDH release assay. Triton-X 100 (0.1%) served as a positive lysis control (P). Results are indicated as a relative percentage to positive lysis control. Data are presented as mean ± SD of three independent experiments (*, P < 0.05; **, P < 0.01; ***, P < 0.001; ****, P < 0.0001; ns, not statistically significant, compared to 0 μg/ml). [file Image_2.jpeg]

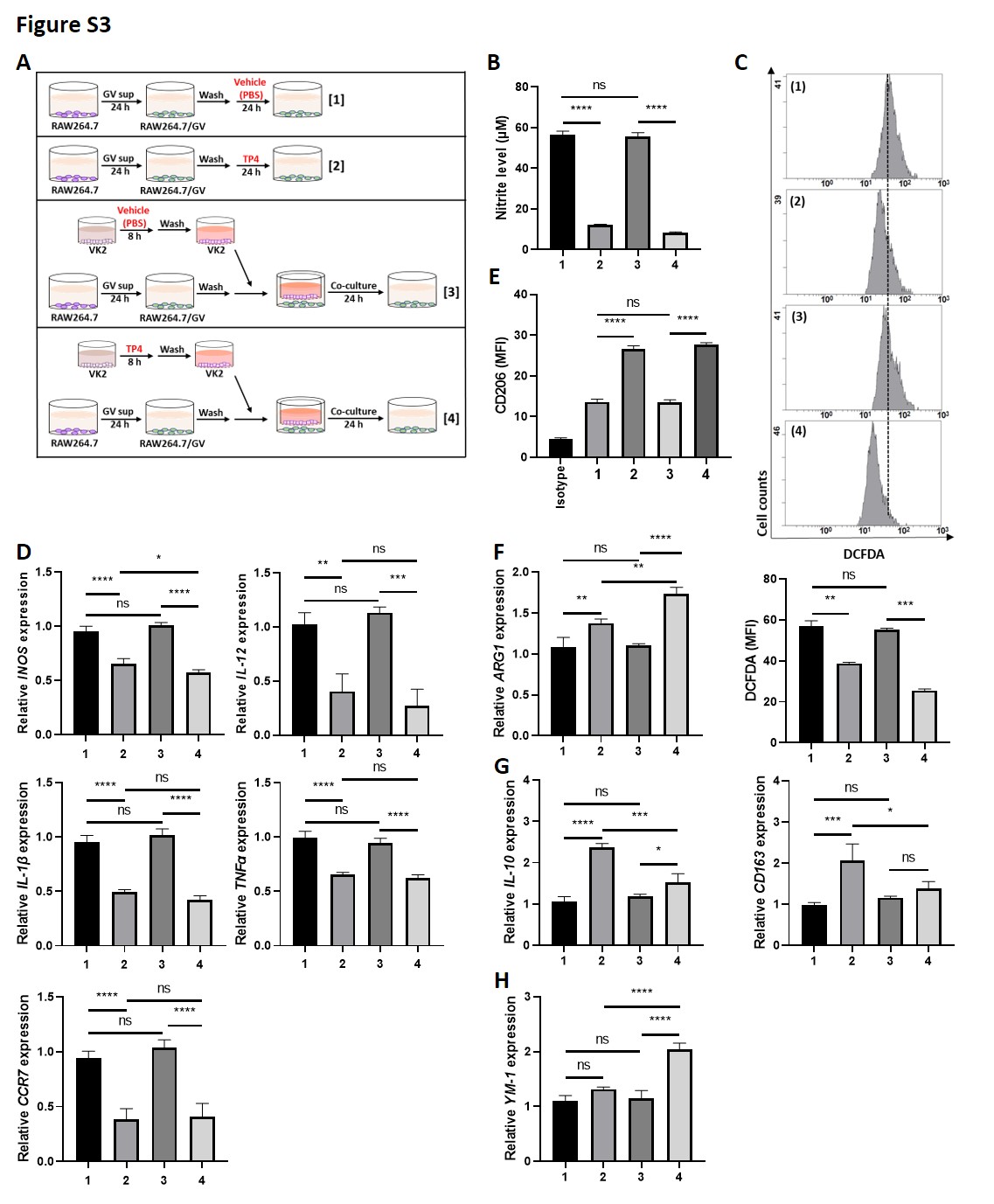

Supplement: Supplementary Figure 3 — TP4 induces M2 phenotypes in RAW264.7/GV cells. (A) Schematic description of the TP4 treatment methods for RAW264.7/GV cells. RAW264.7 cells were stimulated with 10% (v/v) G. vaginalis-free culture supernatants (GV sup) for 24 h. After incubation, RAW264.7/GV cells were washed with PBS and treated with [treatment 1] vehicle (PBS) or [treatment 2] TP4 (7.82 µg/ml) for 24 h or co-cultured with VK2 cells which had been pre-treated with [treatment 3] vehicle (PBS) or [treatment 4] TP4 (7.82 µg/ml) for 8 h and then replaced with fresh culture medium on cell culture inserts. (B) The nitric oxide (NO) levels in RAW264.7/GV cells were assayed by the NO assay. (C) The reactive oxygen species (ROS) levels in RAW264.7/GV cells were measured by DCFDA assay using flow cytometry. The mean fluorescence intensity (MFI) is shown in the bar graphs. (D) Gene expression levels of M1-related genes (INOS, IL-12, CCR7, TNFα, and IL-1β) were measured by qRT-PCR. Data were represented the normalized target gene amount relative to the group of treatment 1. (E) Macrophage surface marker (CD206) was detected by flow cytometry; the MFI is shown. The black bar graph indicates isotype controls. (F) ARG1 expression was measured by qRT-PCR. Data were represented the normalized target gene amount relative to the group of treatment 1. (G) IL-10 and CD163 expression levels were measured by qRT-PCR. Data were represented the normalized target gene amount relative to the group of treatment 1. (H) YM-1 expression was measured by qRT-PCR. Data were represented the normalized target gene amount relative to the group of treatment 1. Data are presented as mean ± SD of three independent experiments (*, P < 0.05; **, P < 0.01; ***, P < 0.001; ****, P < 0.0001; ns, not statistically significant). [file Image_3.jpeg]

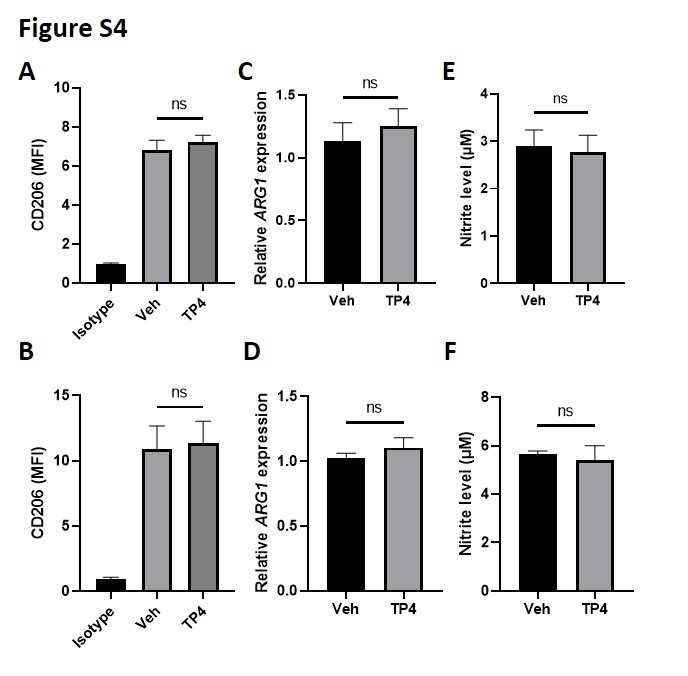

Supplement: Supplementary Figure 4 — TP4 does not affect M1/M2 markers in the absence of GV sup stimulation. PMA-differentiated THP-1 and RAW264.7 cells were treated with vehicle PBS (Veh) or TP4 (7.82 µg/ml) for 24 h. A macrophage surface marker (CD206) was detected by flow cytometry in THP-1 (A) and RAW264.7 (B) cells; the MFI is shown. The black bar graph indicates isotype controls. ARG1 expression levels in THP-1 (C) and RAW264.7 (D) cells were measured by qRT-PCR. Data represent the normalized target gene relative to the vehicle group. Nitric oxide produced by THP-1 (E) and RAW264.7 (F) cells was assayed after TP4 treatment. Data are presented as mean ± SD of three independent experiments (ns, not statistically significant). [file Image_4.jpeg]

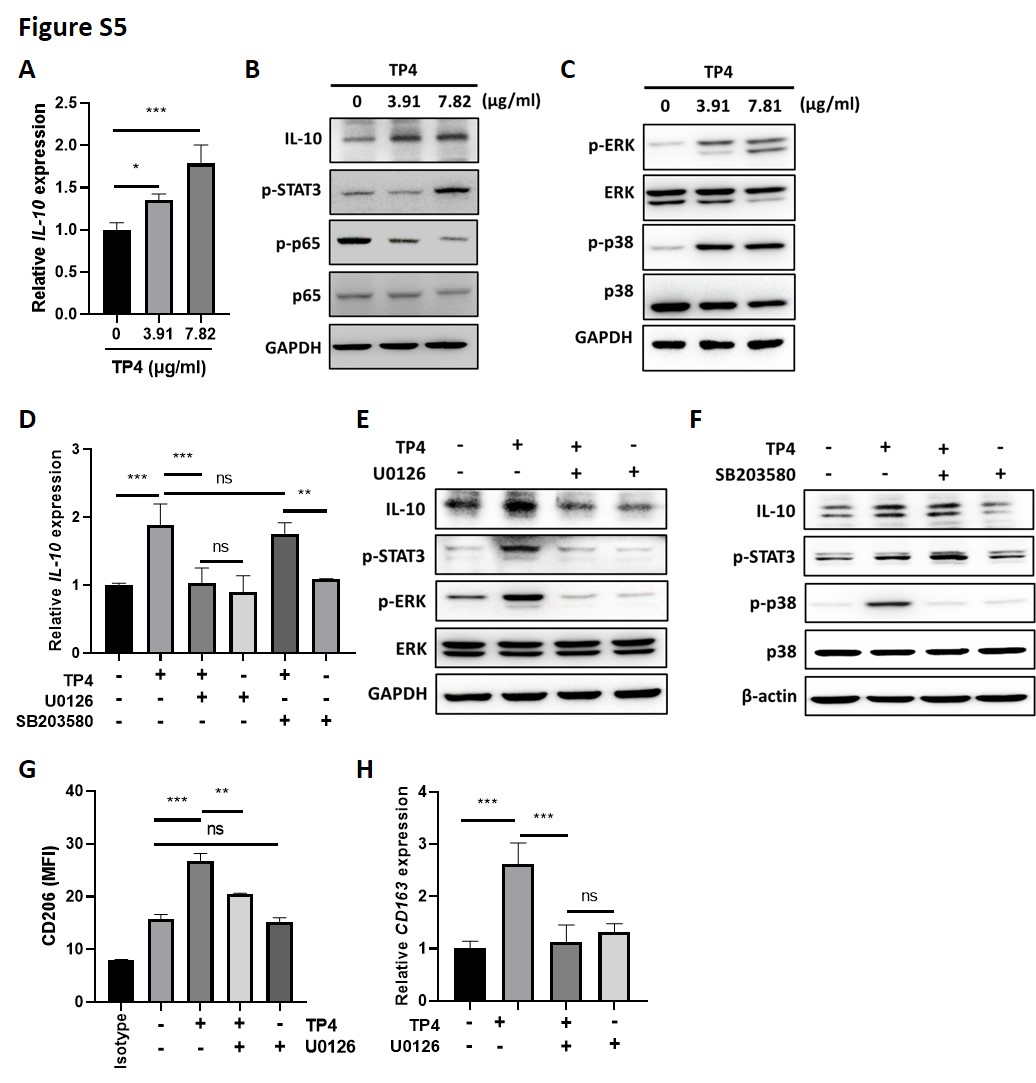

Supplement: Supplementary Figure 5 — TP4 induces IL-10 expression via MAPK/ERK pathway in RAW264.7/GV cells and promotes M2c phenotypes. (A) IL-10 expression after 4 h TP4 (3.91 or 7.82 μg/ml) treatment of RAW264.7/GV cells was measured by qRT-PCR. Data were represented the normalized target gene amount relative to the group of 0 μg/ml. (B) IL-10, and phosphorylated STAT3 (p-STAT3) and NFκB p65 subunit (p-p65) after 4 h TP4 (3.91, 7.82 μg/ml) treatment of RAW264.7/GV cells were detected by immunoblotting. GAPDH was used as an internal control to show equal protein loading. (C) Detection of total and phosphorylated ERK (ERK/p-ERK) and p38 (p38/p-p38) after 4 h TP4 (3.91 or 7.82 μg/ml) treatment of RAW264.7/GV cells by immunoblotting. GAPDH was used as an internal control to show equal protein loading. (D) RAW264.7/GV cells were pre-treated with ERK inhibitor (U0126; 10 μM) or p38 inhibitor (SB203580; 10 μM) for 2 h in serum-free medium. After incubation, TP4 (7.82 μg/ml) was treated for 4 h. IL-10 expression after treatments was measured by qRT-PCR. Data were represented the normalized target gene amount relative to the untreated control group. (E) Detection of IL-10, p-STAT3, p-ERK, and ERK after U0126 (10 μM) and TP4 (7.82 μg/ml) treatments for 4 h in RAW264.7/GV cells by immunoblotting. GAPDH was used as an internal control to show equal protein loading. (F) Detection of IL-10, p-STAT3, p-p38, and p38 after SB203580 (10 μM) and TP4 (7.82 μg/ml) treatments for 4 h in RAW264.7/GV cells by immunoblotting. β-actin was used as an internal control to show equal protein loading. (G) Macrophage surface marker (CD206) was detected by flow cytometry; the mean fluorescence intensity (MFI) is shown. The black bar graph indicates isotype controls. (H) CD163 expression after U0126 (10 μM) and TP4 (7.82 μg/ml) treatments in RAW264.7/GV cells were measured by qRT-PCR. Data were represented the normalized target gene amount relative to the untreated control group. Data are presented as mean ± SD of three independent [file Image_5.jpeg]

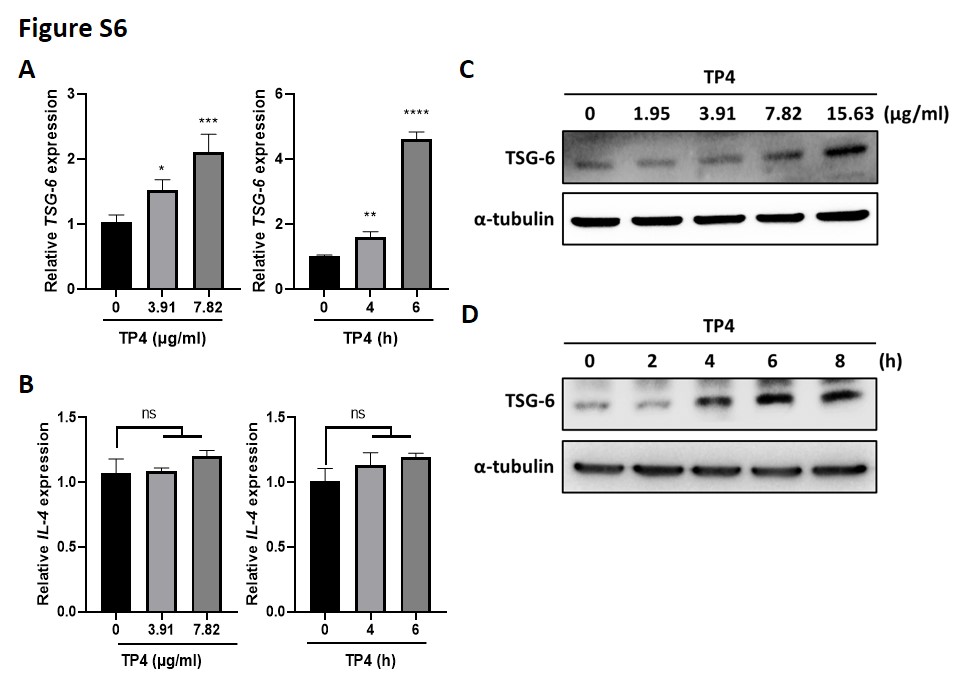

Supplement: Supplementary Figure 6 — TP4 induces TNF-α-stimulated gene 6 (TSG-6) secretion from the endocervical epithelial (End1) cells. (A) TSG-6 expression after TP4 (3.91 or 7.82 μg/ml) treatments for 6 h (left) and 7.82 μg/ml TP4 treatments for 0, 4 or 6 h (right) in End1 cells, measured by qRT-PCR. Data were represented the normalized target gene amount relative to the group of 0 μg/ml. (B) IL-4 expression after TP4 (3.91 or 7.82 μg/ml) treatments for 6 h (left) and 7.82 μg/ml TP4 treatment for 0, 4 or 6 h (right) in End1 cells, measured by qRT-PCR. Data were represented the normalized target gene amount relative to the group of 0 μg/ml. Data are presented as mean ± SD of three independent experiments (*, P < 0.05; **, P < 0.01; ***, P < 0.001; ****, P < 0.0001; ns, not statistically significant, compared to 0 μg/ml). (C) Detection of TSG-6 expression in End1 cells after treatment of TP4 (1.95, 3.91, 7.82 or 15.63 μg/ml) for 6 h by immunoblotting. α-tubulin was used as an internal control to show equal protein loading. (D) Detection of TSG-6 expression in End1 cells after 7.82 μg/ml TP4 treatment for 0, 2, 4, 6 or 8 h by immunoblotting. α-tubulin was used as an internal control to show equal protein loading. [file Image_6.jpeg]

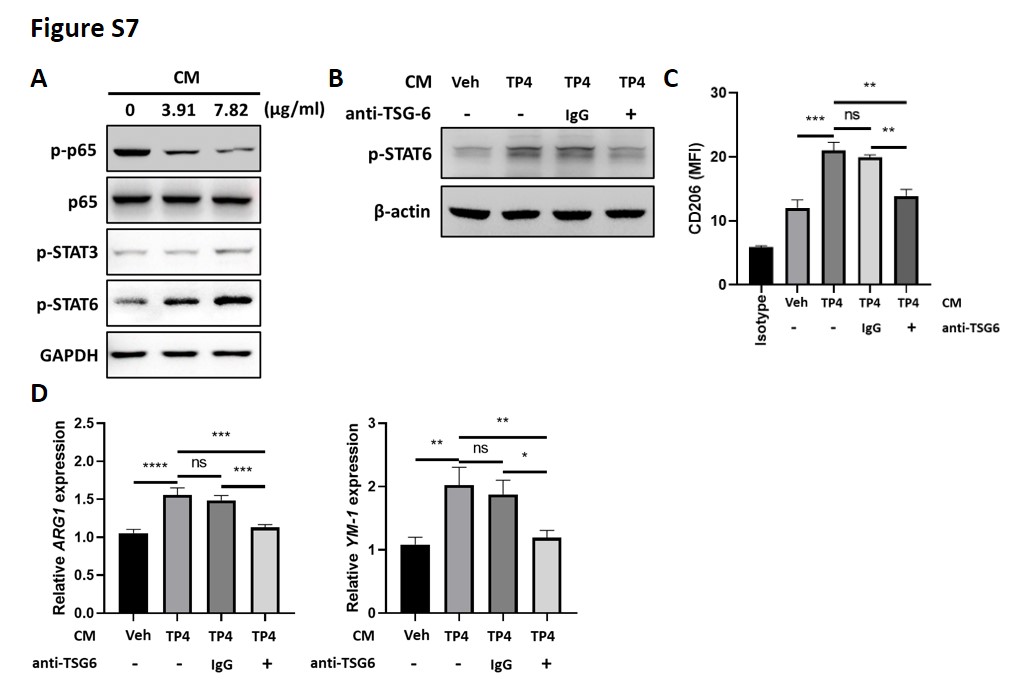

Supplement: Supplementary Figure 7 — TP4-induced TNF-α-stimulated gene 6 (TSG-6) secretion from VK2 cells is required for STAT6 activation in RAW264.7/GV cells. (A) Detection of phosphorylated NFκB p65 subunit (p-p65), STAT3 (p-STAT3), and STAT6 (p-STAT6) after 1 h treatment with the conditioned medium (CM) from TP4 (3.91 or 7.82 μg/ml)-treated VK2 cells to RAW264.7/GV cells by immunoblotting. β-actin was used as an internal control to show equal protein loading. (B) The CM from vehicle (Veh; PBS)- or TP4-treated VK2 cells were incubated with TSG-6 neutralizing antibody (anti-TSG6) or IgG control for 1 h and then used to treat RAW264.7/GV cells. After incubation, p-STAT6 in RAW264.7/GV cells was detected. GAPDH was used as an internal control to show equal protein loading. (C) Macrophage surface marker (CD206) was detected by flow cytometry after CM treatments. The mean fluorescence intensity (MFI) is shown. The black bar graph indicates isotype controls. (D) ARG1 (left) and YM-1 (right) expression levels were measured after treatments by qRT-PCR. Data were represented the normalized target gene amount relative to the vehicle-treated group (lane 1). Data are presented as mean ± SD of three independent experiments (*, P < 0.05; **, P < 0.01; ***, P < 0.001; ****, P < 0.0001; ns, not statistically significant). [file Image_7.jpeg]
