# Supplementary material for: Tilapia Piscidin 4 (TP4) Reprograms M1 Macrophages to M2 Phenotypes in Cell Models of Gardnerella vaginalis-Induced Vaginosis
Source: Front Immunol. 2021 Dec 2;12:773013. doi: 10.3389/fimmu.2021.773013 (PMC8674419; doi:10.3389/fimmu.2021.773013)
Supplement: Supplementary file 8 [file Table_1.docx]

**Supplementary Table 1.** Primers used in the qRT-PCR analyses.

| **Target Gene** | **Primer Sequences**  **(5’-3’)** | **GenBank Number** | **Product Length (bp)** |
| --- | --- | --- | --- |
| **Human** |  |  |  |
| *INOS* | F- ATGCCCGATGGCACCATCAGA  R- TCTCCAGGCCCATCCTCCTGC | NM_000625.4 | 394 |
| *IL-12* | F- GCCACAAAAATCCTCCCTTG  R- CTCCCTAGTTCTTAATCCAC | NM_001354583.2 | 217 |
| *CCR7* | F- ACAGCCTTCCTGTGTGGTTT  R- ATGATGGAGTACATGATAGG | NM_001301714.2 | 191 |
| *CXCL10* | F- GAAATTATTCCTGCAAGCCAATTT  R- TCACCCTTCTTTTTCATGTAGCA | NM_001565.4 | 67 |
| *TNFα* | F- GCCCCAATCCCTTTATTACC  R- CACATTCCTGAATCCCAGGT | NM_000594.4 | 145 |
| *IL-1β* | F- CAGTGGCAATGAGGATGACTTG  R- AGTGGTGGTCGGAGATTCGT | NM_000576.3 | 125 |
| *ARG1* | F- GTTTCTCAAGCAGACCAGCC  R- GCTCAAGTGCAGCAAAGAGA | NM_000045.4 | 149 |
| *IL-10* | F- GAGGCTACGGCGCTGTCA  R- TCCACGGCCTTGCTCTTG | NM_001382624.1 | 57 |
| *SPHK1* | F- CATCCAGAAGCCCCTGTGTAG  R- GTCTTCATTGGTGACCTGCTCAT | NM_001142601.2 | 100 |
| *YM-1* | F- CCAGTGCTGCTCTGCATACA  R- ATGCCGTAGAGCGTCACATC | NM_001276.4 | 189 |
| *RELMα* | F- CGTCCTCTTGCCTCCTTCTC  R- ACAAGCACAGCCAGTGACAG | NM_032579.3 | 227 |
| *MMP9* | F- GTGGCGGCGCATGAGT  R- AGGTGCCGGATGCCATT | NM_004994.3 | 134 |
| *ZO-1* | F- AGAAGGATGTTTATCGTCGCATT  R- CCAAGAGCCCAGTTTTCCAT | NM_001330239.4 | 164 |
| *IL-4* | F- CCGTAACAGACATCTTTGCTGCC  R- GAGTGTCCTTCTCATGGTGGCT | NM_001354990.2 | 209 |
| *TSG-6* | F- TCATGTCTGTGCTGCTGGATG  R- GGGCCCTGGCTTCACAA | NM_007115.4 | 67 |
| *GAPDH* | F- ACAGTCAGCCGCATCTTCTT  R- GACAAGCTTCCCGTTCTCAG | NM_001357943.2 | 205 |
| **Mouse** |  |  |  |
| *INOS* | F- CCAAGCCCTCACCTACTTCC  R- CTCTGAGGGCTGACACAAGG | NM_001313922.1 | 127 |
| *IL-12* | F- AGTGACATGTGGAATGGCGT  R- CAGTTCAATGGGCAGGGTCT | NM_001303244.1 | 133 |
| *CCR7* | F- AAAGCACAGCCTTCCTGTGT  R- AGTCCACCGTGGTATTCTCG | NM_001301713.1 | 115 |
| *IL-6* | F- CCTTCCTACCCCAATTTCCAA  R- AGATGAATTGGATGGTCTTGGTC | NM_031168.2 | 89 |
| *TNFα* | F- ACGGCATGGATCTCAAAGAC  R- AGATAGCAAATCGGCTGACG | NM_001278601.1 | 138 |
| *IL-1β* | F- AGTTGACGGACCCCAAAAG  R- AGCTGGATGCTCTCATCAGG | NM_008361.4 | 75 |
| *ARG1* | F- CAGAAGAATGGAAGAGTCAG  R- CAGATATGCAGGGAGTCACC | NM_007482.3 | 250 |
| *IL-10* | F- CCAGTTTTACCTGGTAGAAG  R- TGTCTAGGTCCTGGAGTCCA | NM_010548.2 | 324 |
| *CD163* | F- CCTGGATCATCTGTGACAACA  R- TCCACACGTCCAGAACAGTC | NM_001170395.1 | 67 |
| *YM-1* | F- AGAAGGGAGTTTCAAACCTGGT  R- GTCTTGCTCATGTGTGTAAGTGA | NM_009892.3 | 109 |
| *GAPDH* | F- ACAGTCAGCCGCATCTTCTT  R- GACAAGCTTCCCGTTCTCAG | NM_008084.3 | 248 |
